# Sensilla Trichoidea-Inspired, High-Temperature, and Omnidirectional Vibration Perception Based on Monolayer Graphene

**DOI:** 10.1007/s40820-025-02029-z

**Published:** 2026-01-12

**Authors:** Yuning Li, Danke Chen, Xiaoqiu Tang, Peizhi Yu, Jingye Sun, Xue Li, Qing You, Mingqiang Zhu, Chang Gao, Linan Li, He Tian, Tao Deng

**Affiliations:** 1 State Key Laboratory of Advanced Rail Autonomous Operation, Beijing Jiaotong University, Beijing, People’s Republic of China; 2 School of Electronic and Information Engineering, Beijing Jiaotong University, Beijing, People’s Republic of China; 3 Department of Precision Instrument, Tsinghua University, Beijing, People’s Republic of China; 4 School of Integrated Circuits and Beijing National Research Center for Information Science and Technology (BNRist), Tsinghua University, Beijing, People’s Republic of China

**Keywords:** Bioinspired, 3D, Graphene, Vibration perception, Monolithic integration

## Abstract

With the convergence of sensor technology, artificial intelligence, and the Internet of Things, intelligent vibration monitoring systems are undergoing transformative development. This evolution imposes stringent demands on the miniaturization, low power consumption, high integration, and environmental adaptability of transducers. Graphene, renowned for its superlative physicochemical attributes, holds significant promise for application in micro- and nanoelectromechanical systems (M/NEMS). However, the inherent central symmetry of graphene restricts its utility in piezoelectric devices. Inspired by the sensilla trichoidea of spiders, a three-dimensional (3D) cilia-like monolayer graphene omnidirectional vibration transducer (CGVT) based on a stress-induced self-assembly mechanism is fabricated, demonstrating notable performance and high-temperature resistance. Furthermore, 3D vibration vector decoding is realized via an omnidirectional decoupling algorithm based on one-dimensional convolutional neural networks (1DCNN) to achieve precise discrimination of vibration directions. The 3D bionic vibration-sensing system incorporates a spider web structure into a bionic cilia MEMS chip through a gold wire bonding process, enabling the realization of three distinct mechanisms for vibration detection and recognition. In particular, these devices are manufactured using silicon-based semiconductor processing techniques and MEMS fabrication methodologies, leading to a substantial reduction in the dimensions of individual components compared to traditional counterparts.

**Supplementary Information:**

The online version contains supplementary material available at 10.1007/s40820-025-02029-z.

## Highlights

- Bioinspired MEMS vibration perception: The monolithic integration of three-dimensional semicircular biomimetic ‘cilia’-structured vibration transducer arrays based on monolayer graphene was achieved by a controlled stress-driven self-assembly technique.
- High Performance: The 3D vibration transducer array enables real-time, high-performance (87.95 pC g^−1^), and wide-range vibration monitoring (1 Hz–10 kHz, 0–1120 g) under dynamic loading, while achieving omnidirectional vibration signal acquisition and decoupling.
- Resistant to high temperatures: Stable vibration responses at ultrahigh temperatures up to 800 °C are achieved with merely a 20-nm-thick Si_3_N_4_ protective coating.

**Supplementary Information:**

The online version contains supplementary material available at 10.1007/s40820-025-02029-z.

## Graphical abstract

**Figure:**
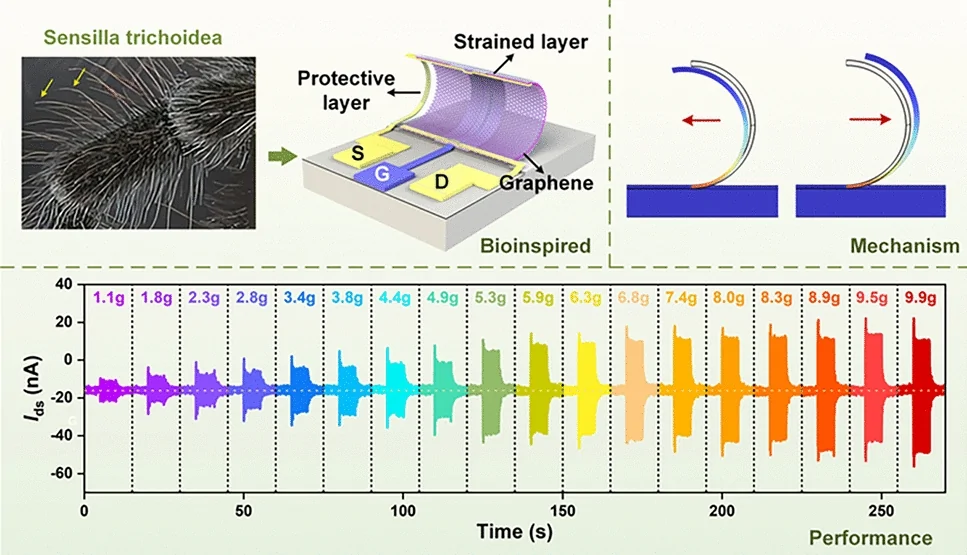

## Introduction

Vibration perception, being an essential component of contemporary measurement and monitoring technology, demonstrates invaluable functional significance in critical domains such as equipment condition assessment [1], structural health monitoring [2], dynamics research [3], and industrial optimization design [4]. The profound integration of sensor technology with artificial intelligence, the Internet of Things, and other cutting-edge technologies is accelerating the intelligent advancement of the vibration monitoring industry and fostering multidisciplinary innovation. Current requirements for vibration sensors emphasize miniaturization, low power consumption, high integration capability, robust resistance to overloads, and adaptability to harsh environmental conditions. Addressing these challenges necessitates improvements and innovations in materials and structures, coupled with the exploration of novel principles. Monolayer graphene emerges as an unparalleled candidate for micro-nano-electromechanical systems (MEMS/NEMS), due to its atomic thickness, exceptional mechanical properties, and outstanding electrical conductivity [5–7]. Regrettably, the inherent centrosymmetry of graphene fundamentally restricts its potential for piezoelectric applications [8]. Flexoelectricity, defined as the strain-gradient-induced polarization, represents a micro/nanoscale force-electric coupling phenomenon that circumvents symmetry constraints and Curie temperature limitations, opening new possibilities for nanomaterial-based functional devices [9]. Notably, flexoelectric enhancement becomes more pronounced with reduced feature sizes, enabling advanced electromechanical transduction in monolayer graphene [10–12]. In previous groundbreaking research, the achievement of flexoelectricity or exceptional two-dimensional (2D) piezoelectricity necessitated intricate techniques like graphene functionalization, distinctive structures, and suspension [13–15]. Although suspended monolayer graphene exhibits exceptional deformation resistance, stress gradients inevitably induce slip, flexural instability, and fracture failure, severely compromising fabrication yield.16 Moreover, generating measurable electrical parameter variations under dynamic microscopic load remains challenging, confining flexoelectricity evaluation in 2D materials to theoretical models and limited experimental characterization [17, 18].

**Fig. 1:**
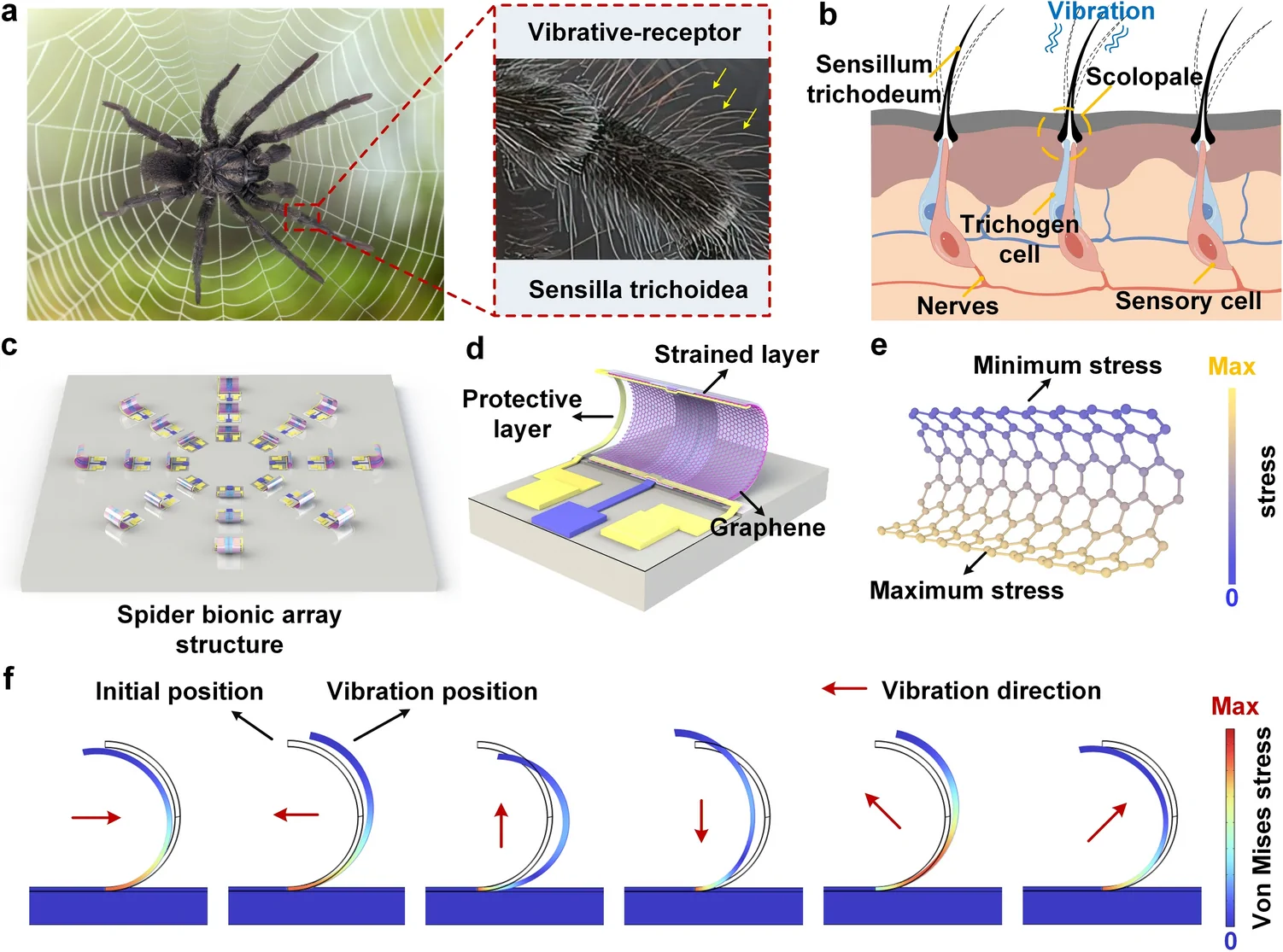
Design and concept. (**a**) Vibration sensilla system of spider. The illustration depicts a partially magnified spider leg, replete with numerous sensilla trichoidea. (**b**) Schematic diagram of a vibration sensilla system. (**c**) Schematic diagram of a spider bionic array structure. (**d**) Schematic diagram of the 3D self-supporting cilia-like monolayer graphene omnidirectional vibration transducer (CGVT). (**e**) Schematic of the stress distribution in a monolayer graphene on a 3D mimetic structure. (**f**) FEM simulated stress distributions of 3D mimetic cilia-like structure under various vibration directions

In the nature, spiders employ intricate webs as precision instruments to locate prey through tiny vibrations transmitted via its silk threads [19]. This extraordinary vibration perception originates from the sophisticated sensilla trichoidea system, formed by arrays of densely distributed bristles on spider legs, as shown in Fig. 1a [20]. These sensory organs mediate environmental perception and stimulus response through coordinated nervous system interactions [21]. As illustrated in Fig. 1b, sensilla trichoidea comprise external sensillum trichodeum and internal components including trichogen cells, sensory cells, and associated neural networks. The underlying vibration sensing mechanism involves external mechanical stimuli inducing flexion of setae, propagating oscillation through trichogen cells to sensory cells, ultimately generating neuronal action potentials through mechanoelectrical conversion. Emulating this hierarchical architecture, we developed a 3D self-supporting cilia-like monolayer graphene omnidirectional vibration transducer (CGVT) capable that enables self-powered sensing. Our proposed stress-layer self-assembly technique transforms planar 2D graphene field-effect transistors (GFETs) into 3D semicircular bionic “cilia” structures (Fig. 1d), simultaneously breaking the structural symmetry of the intrinsic graphene crystal and establishing novel electromechanical coupling. The resultant 3D CGVT arrays with tunable curvature radii and stiffness (Fig. 1c) enable omnidirectional vibration detections. Figure 1e schematically illustrates the stress distribution in a single-layer graphene on a 3D mimetic structure, highlighting maximum stress near the fixed end and minimum stress near the free end, with significant variations observed along the circumferential direction. This reveals the design of specialized transverse electrodes in the CGVT, differing from conventional FETs. The electrodes are positioned at stress maxima and minima to capture a greater amount of polarization charge and optimize the response. Under the vibration, oppositional compression/tension of cilia-like bionic structures (CLBS) generates direction-dependent strain heterogeneity, as confirmed by finite element simulations (Fig. 1f). To ensure high-temperature stability, we integrate ultra-thin silicon nitride nano-protection to isolate graphene from the external environment such as oxygen and water molecules, and protect it from oxidative degradation. Complementing this, we developed a streamlined 1D convolutional neural network for rapid vibration direction decoupling through time-series analysis. Remarkably, the hybrid fabrication combining silicon semiconductor processing technology and MEMS manufacture techniques yields lightweight and highly integrated devices suitable for space-constrained platforms. This methodology unlocks new potential for next-generation vibration transducers with enhanced integration, performance, and intelligence across symmetric and asymmetric thin-film material systems.

## Experimental Section

### Materials

Trivial Transfer Graphene was purchased from Nanjing XFNANO Materials Tech Co.,Ltd. Two-component inorganic adhesive was purchased by Shenzhen Sinwe Electronic Materials Co., Ltd.

### Preparation of Vibration Transducer (Devices/Arrays)

The CGVTs are inspired by a stress-induced self-assembly process to render 3D non-closed microtubule structures, creating conditions for vibration sensing (Fig. S1). First, the (100) crystalline phase of the 4-inch silicon substrate was cleaned with acetone, alcohol, and deionized water. Then, a sacrificial layer Al (~ 50 nm) was magnetron sputtered on the wafer. Dual stress layers (SiN_x_) with opposite stresses of tension and extension were deposited on the sacrificial layer by the Mixed Frequency Nitride PECVD System. Subsequently, the buried gate electrode (Cr/Au, 10/50 nm) and source-drain electrodes (Ti/Pt, 10/50 nm) appeared by magnetron sputtering, and the two layers were obstructed by a SiO_2_ (30 nm) dielectric layer deposited by PECVD. The CVD-grown monolayer graphene was transferred to the substrate and repeatedly soaked in acetone to remove the polymethyl methacrylate (PMMA), which was followed by a Si_3_N_4_ (20 nm) protective layer tightly grown on the graphene surface by ICPECVD, immediately followed by etching away the areas outside the pattern with ICP-F and alpha plasma. As such, planar transducers with a protective layer have been obtained. Finally, the CGVTs could be fabricated by etching the Al sacrificial layer using a mixed solution of FeCl_3_·6H_2_O and HCl and directional release of the double stress layer with acetone. All the steps were accurately patterned by a photolithographic process to enable the preparation of large-scale arrays. The yield statistics of devices are shown in Fig. S2.

### Characterization

The structure of the CGVT was characterized using optical microscopy (Olympus, MX61). Furthermore, the scanning electron microscope (HITACHI, SU8220) demonstrated the dimensional and surface morphology of individual devices and arrays in detail. Raman (HORIBA, HR Evolution) investigated the material properties of the graphene functional layer before and after it was protected and curled.

### Device Packaging

For vibration performance testing, two packaging methods were designed for 3D CGVT chips in this work. At room temperature, the 3D CGVT die was attached to a Printed Circuit Board (PCB) that could be fixed to a standard vibration exciter for bonding to the Edge Connector, and the signal was then led out by soldering extension wires. Further, in order to fully utilize the potential of the 3D CGVT device at high temperatures, a ceramic encapsulated housing (Al_2_O_3_, resistant to ~ 1750 °C) that can be mechanically fixed to a standard vibrational excitation source was designed and fabricated. The 3D CGVT die was fixed by the high-temperature resistant glue (two-component inorganic adhesive, resistant to ~ 1730 °C), and the source-drain electrodes were connected to the metal pins through gold wire bonding, and then led out to the response collection side through an extended nickel wire with glass fiber covering. The encapsulated chip as a whole could withstand temperatures of at least 800 °C.

### Vibration Transducer Performance Testing

For different frequency ranges, the Type 4827 modal exciter from HBK corporation was used in the range of 1–5 Hz, while the Type 4808 vibration exciter was applied to create the excitation signal from 5 Hz to 10 kHz. The power amplifier and charge amplifier linked with the exciter were 2712 and 2692, respectively. The Dynamic Parameter Detection System (DP730) was used to transmit and collect charge changes for comparative calibration (Fig. S6). For acceleration range testing, the Type 4808 standard shaker was still suitable for low accelerations, while the Pneumatic Standard Shock Table (9525C) was utilized to provide shock-type high accelerations. The high-temperature environment was created by the high-temperature furnace and its control system (GW-1200A), which could provide a temperature range of 20 °C to 1200 °C. The acquisition and monitoring of current signals during the above tests were all realized with a Precision source/Measurement Unit (B2911B).

## Results and Discussion

### Metrology and Characterization

**Fig. 2:**
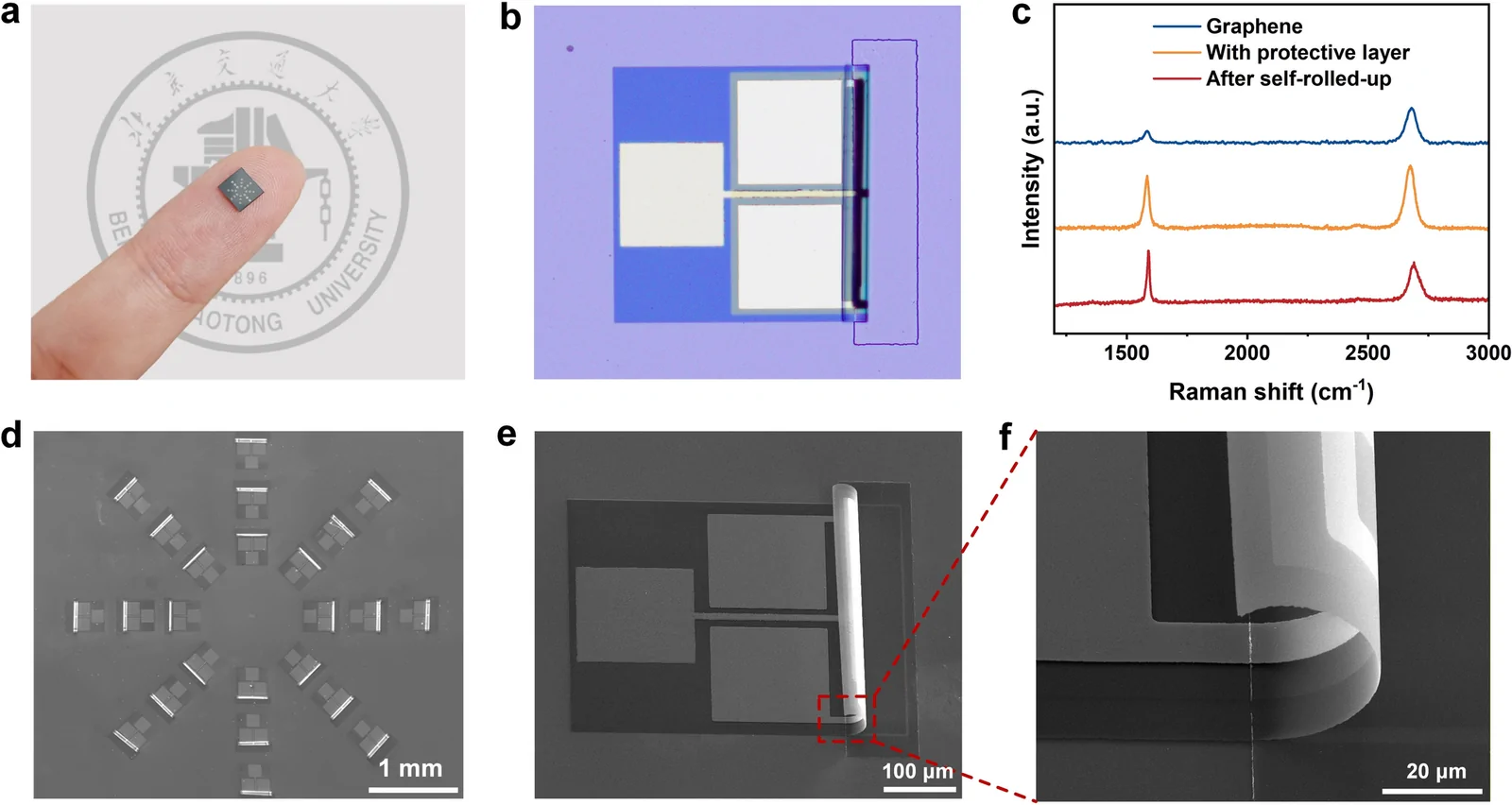
Metrology and characterization. (**a**) Monolithic integrated petal-like CGVT array stands on the finger. (**b**) Optical microimage of the CGVT. (**c**) Raman spectra of the conductive channel of the 2D device with and without protective layer and after stress-induced self-assembly. (**d**) SEM image of the petal-like CGVT array. (**e**) SEM side view image of the CGVT. (**f**) The enlarged view of the CLBS

To determine the bionic structure and the presence of graphene in the CGVT, a representative sample was subjected to various characterization techniques. The dimensions of the single CGVT are about 0.373 mm × 0.3 mm, and the dimensions of the integrated monolithic petal-like CGVT array are about 4 mm × 4 mm (Fig. S3). The monolithic integrated petal-like CGVT array is depicted in Fig. 2a, positioned on a finger. The top-view optical microscopy image of the CGVT in Fig. 2b reveals the internal transverse electrodes, which are clearly visible due to the transparency of both the silicon nitride film and the graphene. To further validate the impact of the guarding process and the self-rolled-up process on the graphene, Raman spectroscopy measurements were conducted, as depicted in Fig. 2c. The G and 2D peaks of the graphene are located at 1589.4 and 2683.7 cm^−1^, respectively. After the protection process and the stress-induced self-assembly process, the two characteristic peaks remain present and are highly pronounced. The top view image of a monolithic integrated petal-like CGVT array obtained through scanning electron microscopy (SEM) is presented in Fig. 2d. The entire array comprises three concentric circles, each containing eight identical devices. From the innermost to the outermost circle, the arc lengths of these devices are 75, 100, and 125 μm, respectively. Different device sizes are employed to achieve varying vibration detection ranges, thereby enhancing the overall limit of detection. From the SEM side-view image of a single CGVT (Fig. 2e) and the enlarged view of the CLBS in Fig. 2f, the curved cilia-like structure is well-shaped and presents a self-supporting state at rest. Additional pre-/post-assembly SEM comparisons are provided in Fig. S4.

### Vibration Measurements and Mechanism Analysis

**Fig. 3:**
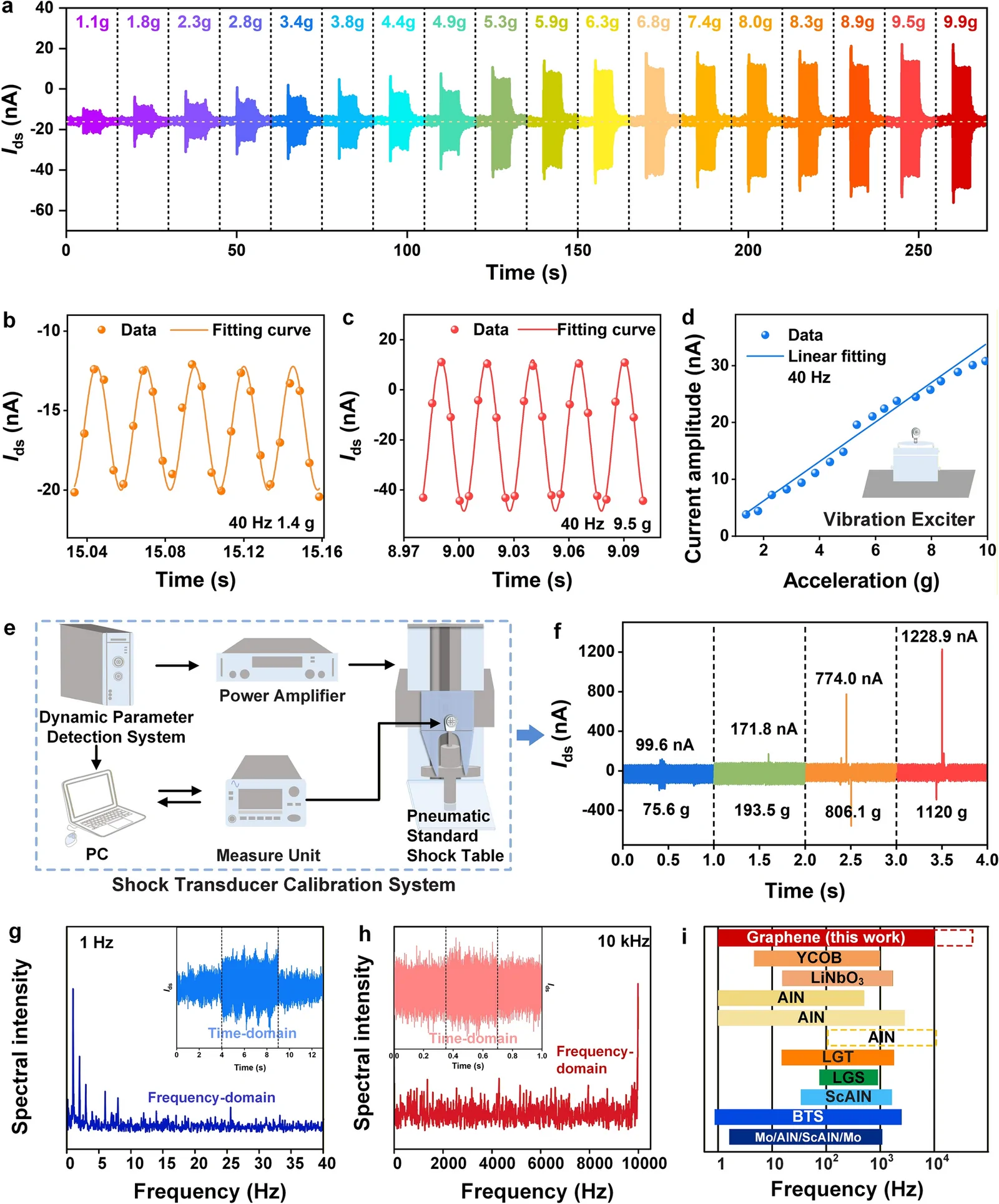
Room temperature response. (**a**) Transient current response of the CGVT under vibration excitation at a frequency of 40 Hz with varying levels of acceleration (1 g = 9.8 m s^−2^). (**b**–**c**) Amplified transient current response of the CGVT under vibration excitation with vibration frequency of 40 Hz and accelerations of 1.4 g and 9.5 g, respectively. The dots represent the data points obtained from test sampling, while the curves represent the fitted curves. (**d**) Response current amplitude as a function of acceleration. The straight line represents a linear fit to the data points. (**e**) Schematic diagram of the experimental setup for the shock transducer calibration system. (**f**) Transient current response of the CGVT under single pulse shock excitation with varying levels of acceleration. (**g**–**h**) Frequency-domain response of the CGVT under vibration excitation at frequencies of 1 Hz and 10 kHz, respectively. The insets display the time-domain response plots of the corresponding device. (**i**) Comparison of effective frequency response range for different families of materials. The dashed boxes represent theoretical values

To systematically evaluate the vibration response of the CGVTs, they were wire bonded and packaged. Low-amplitude accelerations were applied via a standard vibration exciter, with reference measurements acquired using a calibrated transducer. Uniformly select short-circuit current measurement to evaluate the vibration response of CGVTs (Fig. S5). The output transient source-drain currents (*I*_ds_) of the device under vibration excitation with different accelerations from 1.1 to 9.9 g at a vibration frequency of 40 Hz are shown in Fig. 3a. In the absence of vibration excitation, the acquired current signal demonstrated a disordered noise pattern with a peak-to-peak noise measured current approximately 4 nA (Fig. S7). With the application of vibration excitation, the CGVT elicited a significant response current signal. The results obtained in this study demonstrate a positive correlation between increased acceleration and the amplitude of the output current signal. Figure 3b, c illustrates the amplified transient current responses of the CGVT under vibration excitation with accelerations of 1.4 and 9.5 g, respectively. The response current exhibits a typical sinusoidal variation with a frequency of 40 Hz, which is consistent with the oscillator source. The linearity of the CGVT was subsequently assessed by examining the correlation between the input acceleration and the magnitude of the output response current amplitude (Fig. 3d). The response current of the device exhibits a nearly linear increase with increasing acceleration. Using the comparative method (Fig. S6), the maximum charge sensitivity of the device is up to 87.95 pC g^−1^. The higher acceleration tests were conducted utilizing a shock transducer calibration system, as illustrated in Fig. 3e. The transient current responses of the device under various ultra-high acceleration single pulse vibration excitations are shown in Fig. 3f. The response current values exhibit a positive correlation with the acceleration values across four distinct acceleration excitations (i.e., 75.6, 193.5, 806.1, and 1120 g), demonstrating excellent acceleration test range. The response current reached a remarkable value of 1228.9 nA at 1120 g. The frequency measurement range was explored by conducting vibration tests of the CGVTs at various frequencies from 1 Hz to 10 kHz. The frequency of vibration is determinable in low-frequency vibrations ranging from 1 to 20 Hz by observing a significant change in the period of the response current (Fig. S8). The high-frequency signal undergoes fast Fourier transform (FFT) to convert it from the time domain to the frequency domain, facilitating a more comprehensive analysis of its spectral components. Figure 3gh illustrates the frequency-domain response of the CGVT under vibration excitation at frequencies of 1 Hz and 10 kHz, respectively. As expected, the peak frequency of the response current spectrogram corresponds to the vibration operating frequency, demonstrating the device’s operational capability within a range of 1 Hz–10 kHz. More frequency-domain response plots at other frequencies of vibration can be found in Fig. S9. The charge sensitivity of the CGVT as a function of frequency is presented in Fig. S10. Comparative analysis (Fig. 3i) positions CGVTs superior to other piezoelectric materials [22–31], attributed to the miniaturized design and the exceptional carrier mobility of graphene [32]. The realization of higher-frequency vibration detection is anticipated through the reduction in size and enhancement of graphene quality.

**Fig. 4:**
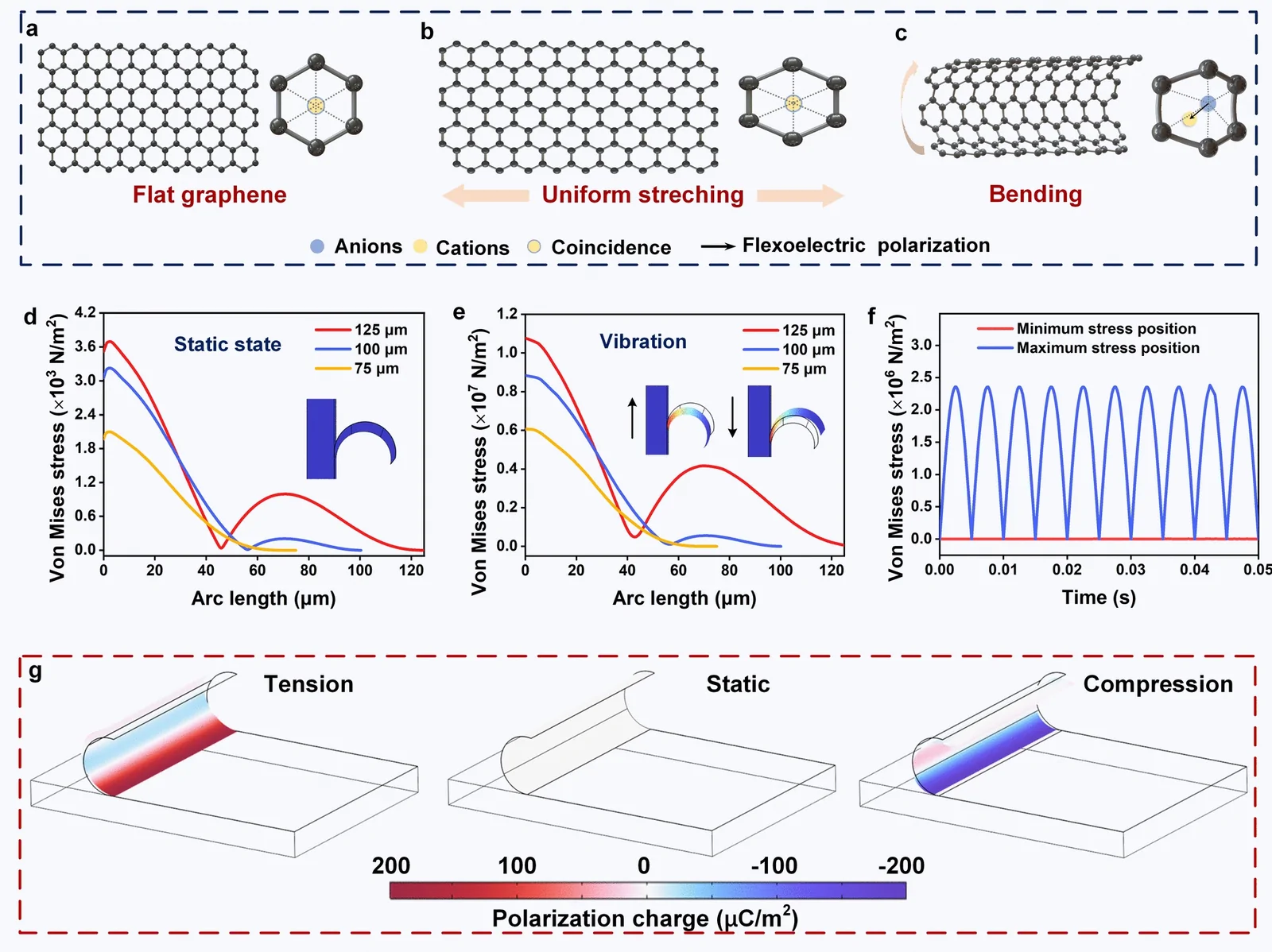
Vibration sensing mechanism of the CGVT. (**a**) Strain-free flat graphene atomic structure. The positive and negative charge geometric centers overlap, resulting in a net dipole moment of zero for the cell. (**b**) Schematic of the graphene atomic structure under uniform stretching. (**c**) Schematic representation depicting the atomic arrangement of graphene in a bent configuration. The presence of a misalignment between the positive and negative ion centers results in the generation of an overall dipole moment. (**d**) Simulated static circumferential stress distribution curves for three different device sizes (75 μm, 100 μm, and 125 μm). (**e**) Simulated circumferential stress distribution curves at a certain moment under vibration. (**f**) Simulated mean values of axial stress at the maximum stress and minimum stress under vibratory conditions as a function of time. (**g**) Simulated polarization charge of the CGVT at tension, static and compression states

These findings suggest a potential mechanism underlying 2D flexoelectricity. It is widely recognized that when certain symmetric bulk 2D materials are reduced to an odd number of layers, the central symmetry is disrupted, leading to the emergence of 2D piezoelectric properties. However, this mechanism does not apply to single-layer graphene due to the presence of an inversion center at its midpoint, as shown in Fig. 4a. As illustrated in Fig. 4b, when graphene is subjected to an uniform stress, despite the positional displacements of the carbon atoms, the geometric centers of positive and negative charges coincide, leading to a net dipole moment of zero for the six-membered carbon ring. When graphene undergoes bending, the redistribution of positive and negative charge centers within the graphene sheet leads to the formation of a net dipole moment in space (Fig. 4c). This pattern could be interpreted from the point of view of orbital hybridization as bending increases Coulomb repulsion and triggers an asymmetric redistribution of graphene π-orbitals [12]. Stress gradients acting on the monolayer graphene become large by size effects, allowing graphene to exhibit a superb 2D flexoelectricity. To better understand the relationship between the change in the stress gradient and the response to vibration, COMSOL simulations were performed (Fig. S11). Figure 4d illustrates the static circumferential stress distribution curves for three different device sizes (75, 100, and 125 μm). The cilia-like bionic structure (CLBS) is affixed to the substrate with fixed constraints. When at rest, gravity induces a nonuniform stress distribution in the circumferential direction of the bionic structure (Fig. 4d). The stress reaches its peak at the interface between the bionic structure and the substrate, and it is minimal in the region aligned tangentially and parallel to the direction of gravity. Subsequently, a fixed duration surface load is applied to the substrate and the device undergoes forced deformation. The CLBS undergoes periodic compression and tension in response to inertia, inducing a periodically varying non-uniform stress gradient in monolayer graphene. The data indicate a plateau where the maximum circumferential stress in the device increases by three orders of magnitude following the application of vibration excitation (Fig. 4e). The CGVT induces a significant stress differential during vibration, and the frequency of this stress fluctuation is precisely aligned with the frequency of the response current variation observed in the experiment (Fig. 4f). The stress gradient, which varies with vibration, induces the production of polarized charges and their redistribution in graphene. Response currents are generated by the collection action of the source-drain electrodes, enabling precise vibration measurements without bias voltage. The simulation results of the polarization charge in the CGVT under tensile, static, and compressive conditions (Fig. 4g) further demonstrate that opposite polarized charges are generated during the contraction and relaxation of ciliary structures, giving rise to vibration-dependent current variations.

### Thermal Safeguarding and Thermal Vibration Analysis

**Fig. 5:**
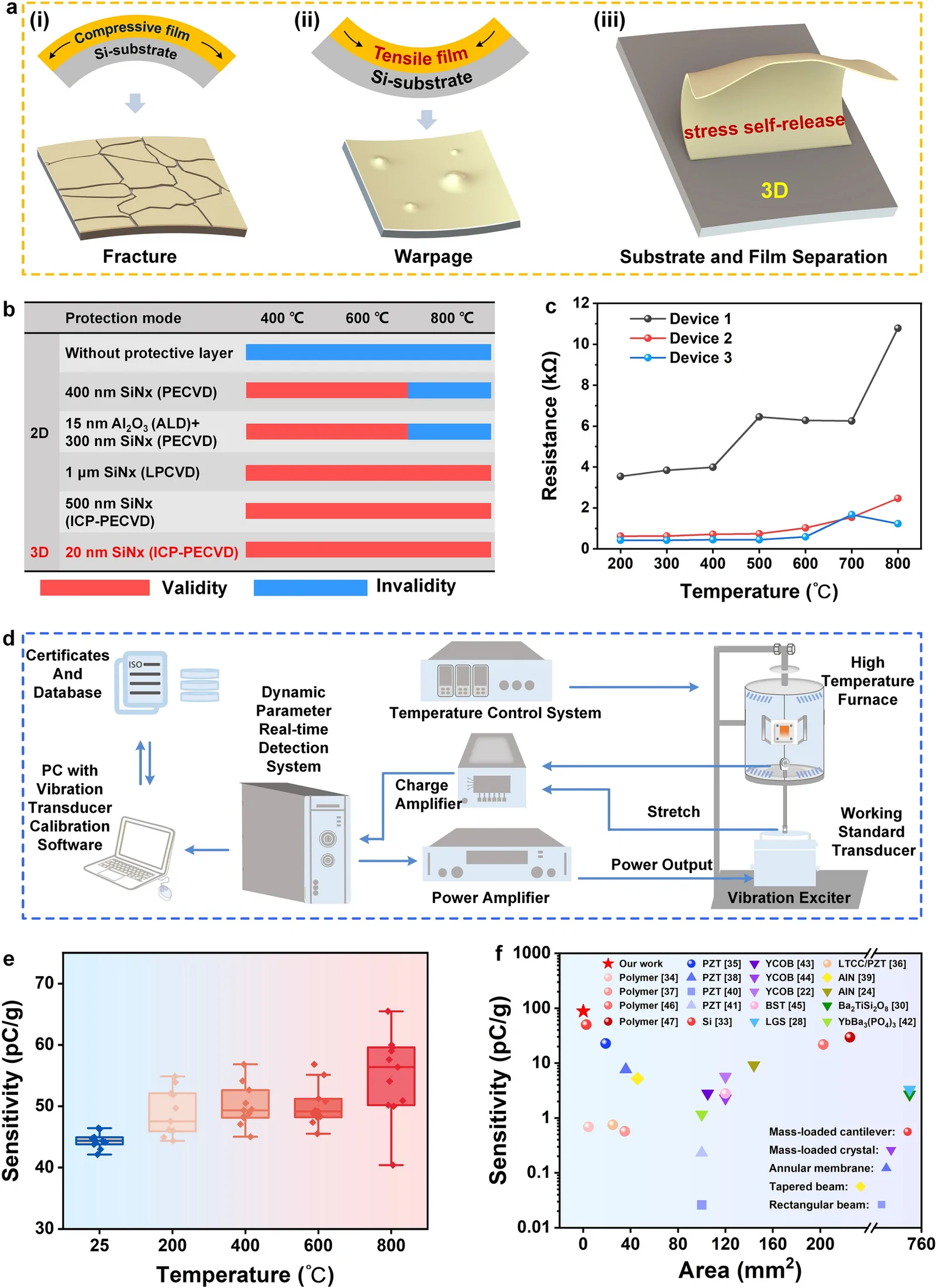
Thermal safeguarding and thermal-vibration response. (**a**) Thermal protection mechanism of 3D devices. (**b**) Comparison of the effectiveness among different protection methods. (**c**) Resistance variation curves of the CGVTs after heat treatment at different temperatures. (**d**) Schematic diagram of the experimental setup for thermal-vibration experiment. (**e**) Sensitivity of the CGVTs with different temperature. (**f**) Reported charge sensitivity versus area for vibration transducers (or accelerometers). The identical symbol shape denotes uniform device structure. This work is marked by the red star

Vibration transducers find application in various industries, energy sectors, aerospace, and other fields that involve operating in challenging environments, particularly those with high temperatures. Graphene’s oxidative degradation in air under high temperature necessitates protective strategies. Here, we introduce a 3D structure-oriented silicon nitride (Si_3_N_4_) nanoencapsulation (Fig. 5a) to address both oxidation and thermal stress challenges. The protective layer technology effectively prevents the oxidation reaction by isolating graphene from oxygen exposure. The residual stresses in the film are compounded by intrinsic stresses, lattice mismatch stresses, and thermal stresses in a high-temperature environment. Film rupture occurs when the internal stress within the film exceeds the critical tensile strength of the material, leading to a failure, as shown in Fig. 5a(i). In contrast, when the film is subjected to excessive compressive stress that cannot be adequately relieved, it can warp or bulge, as decipted in Fig. 5a(ii). Unlike planar devices, the protective layer in 3D structures is spatially separated from the substrate, allowing residual stresses to be relieved through spatial deformation, as illustrated in Fig. 5a(iii). Figure 5b demonstrates a comparative analysis of the withstand temperatures of the devices under various protection modes. Compared to planar protection, the 3D protection layer achieves the same protective effect with a thickness of only 20 nm. Figure 5c shows the resistance variation curve in the devices on the same chip following heat treatment at various temperatures. The ultrathin Si_3_N_4_ film offers effective thermal protection against a significant increase in resistance at temperatures below 800 °C. As the temperature continues to rise, some 3D devices have their stress layers relaxed and expanded into 2D planar devices. When in contact with the substrate, the surface exhibits bubbles and cracks due to the mismatch in thermal expansion coefficients between the material and the substrate, leading to device failure (Fig. S12). To further investigate the vibration characteristics at elevated temperatures, an thermal vibration experimental platform was constructed, as illustrated in Fig. 5d. The charge sensitivity of the transducer under test was determined by direct comparison with a calibrated standard accelerometer. Figure 5e demonstrates the real-time charge sensitivity detection values at various temperatures, indicating that most sensitivities are consistently maintained at a similar level. Benchmarking against state-of-the-art transducers (Fig. 5f) provides a comparison of the charge sensitivity and device area for the reported vibration transducers (or accelerometers), positioning CGVTs as the highest sensitivity massless device (Table S1) [22, 24, 28, 30, 33–47]. At the same time, the size of the device shows a clear advantage, although electrodes dominate the area and there is a room for significant reduction.

### 1DCNN-based Direction Decoupling

**Fig. 6:**
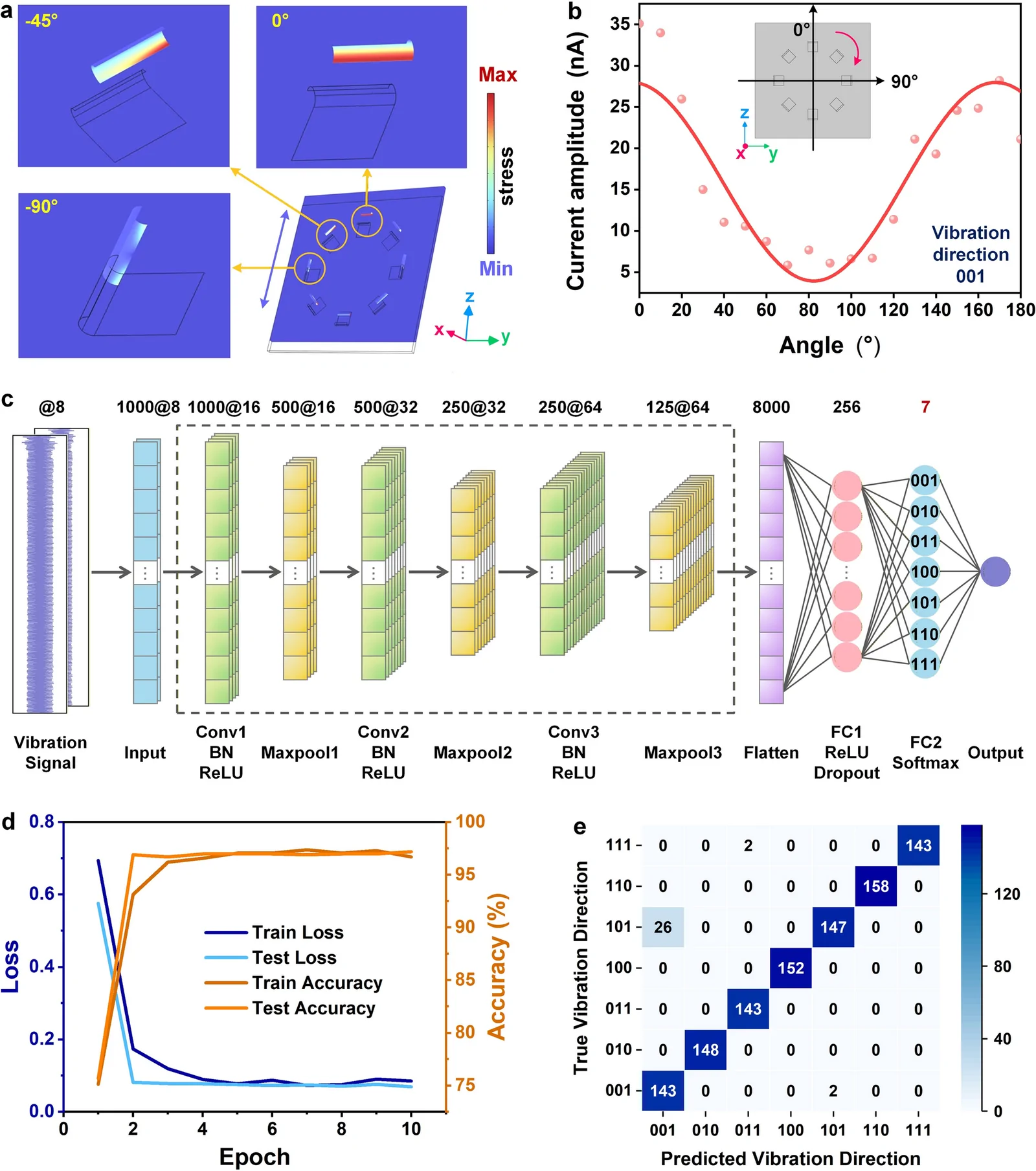
Demonstration of 1DCNN-based vibration direction decoupling. (**a**) Stress simulation under vibration state of sensor arrays. (**b**) Amplitude of device’s current response as a function of rotation angle. (**c**) Architecture of a one-dimensional convolutional neural network for vibration direction decoupling. (**d)** Loss and accuracy rate as a function of the number of training epochs. (**e**) Confusion matrix of the model

Direction-decoding vibration transducers enable critical applications in complex monitoring systems. Due to the unique CLBS, each sensor within the array undergoes a different deformation in response to vibrations in a specific direction. To validate the conjecture, the vertical vibrational stresses on the array were initially simulated using the multiphysics field simulation software COMSOL. As depicted in Fig. 6a, the CLBS generates the highest stress perpendicular to the vibration direction (0°) and the lowest stress when aligned parallel to it (-90°). The omnidirectional vibration experiment was further designed to establish the relationship between the response current amplitude and the various angles of sensor rotation around the center of symmetry with the vibration direction fixed (Fig. 6b). The vibration transducer positioned at various angular locations exhibits distinct characteristics in response to vibration generation, demonstrating a nearly sinusoidal variation.

Using these attributes in conjunction with convolutional neural networks, we can extract feature and pattern recognition from intricate signals. 1DCNN has less computational complexity, is less time-consuming to train, and is well-suited to deal with 1D time-series signals collected from transducers, which can achieve excellent performance in the classification tasks, that is, omnidirectional decoupling in this article. Based on this, we propose an omnidirectional decoupling algorithm based on 1DCNN to realize the classification of the vibration direction of the transducer. The specific model framework and parameters can be found in Fig. 6c. After the vibration signal of current versus time is preprocessed, it is input into the model, and after three convolution and maxpool, the final classification result is output by the second fully connected layer. The input dimensions are 8, which corresponds to eight vibration transducers in a circle. The final layer of the model outputs a 7-dimensional vector representing the predicted probabilities for the seven vibration directions: [001], [010], [011], [100], [101], [110], [111], and then a Softmax activation function is used to convert the output into an effective probability distribution, where the final output of the model is the vibration direction corresponding to the highest probability. The cross-entropy loss function is used to evaluate the error of the predicted output and the true labels. As sketched in Fig. 6d, after 10 epoches, the losses of the training set and the test set no longer decrease and remain stable, with converging around 0.0851 and 0.0691, respectively. For the classification task, accuracy is an important indicator, to evaluate the correct proportion of predictions. The accuracies of the training set and the test set are up to 96.69% and 97.18% after 10 epoches, respectively, demonstrating exceptional performance in identifying the vibration direction of the transducer. In addition, the robustness and generalization ability of the 1DCNN model were further verified in three aspects: simulating harsh environments, data augmentation for ablation experiments, and leave-one-amplitude dataset validation (Figs. S13 and S14). The confusion matrix provides a clear visualization of the recognition rates for seven directions, which is visualized in Fig. 6e, demonstrating high consistency between predicted and true labels.

### Bionic Spider Application

**Fig. 7:**
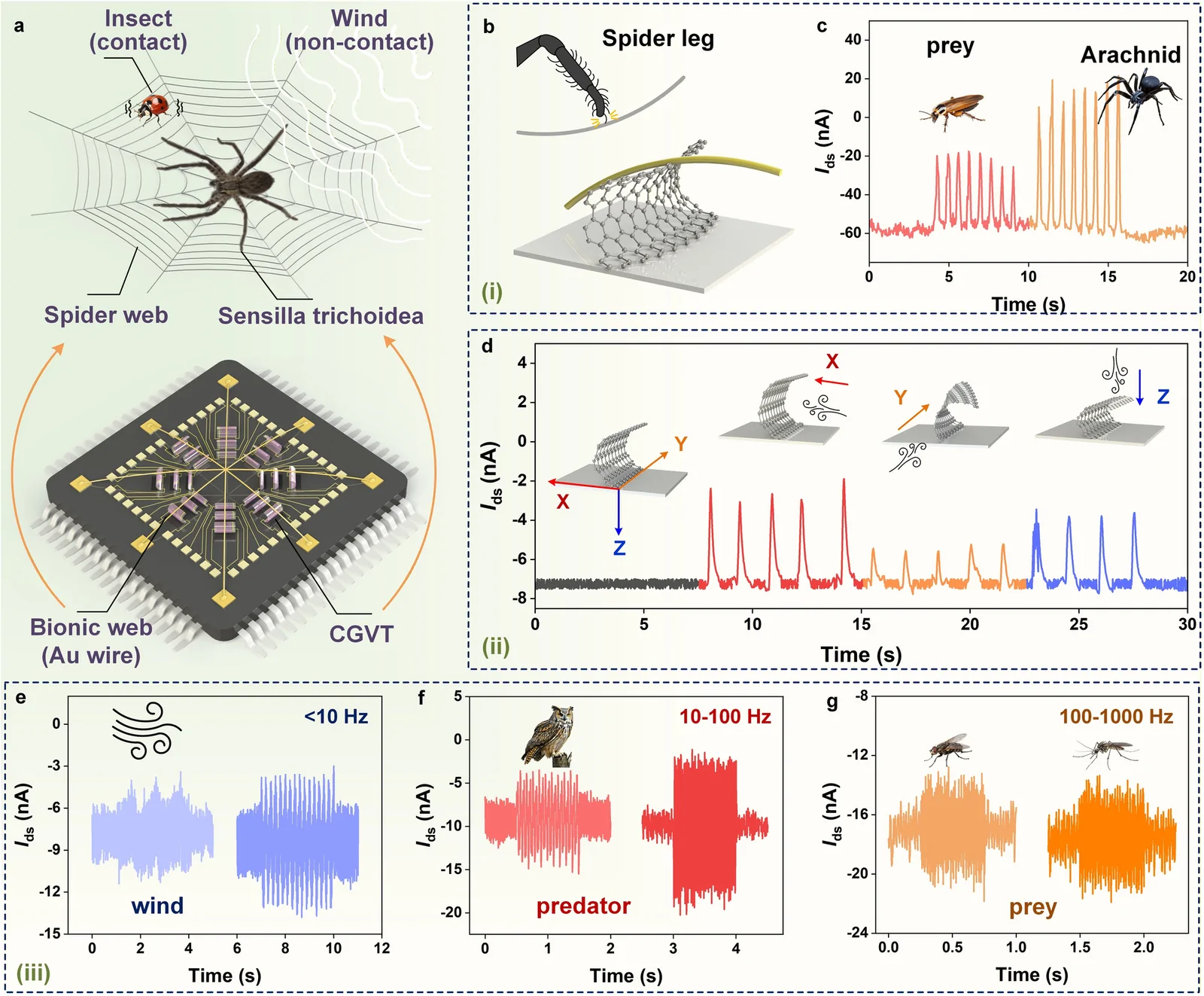
Bionic strategies for vibration sensing of the CGVT. (**a**) Schematic diagram about the CGVT’s applications in vibration monitoring. (**b**) Schematic diagram of suspended silk pulse diagnosis-based vibration sensing. (**c**) Response of the CGVT to varying weights of objects falling onto a bionic spider web. (**d**) Response of the CGVT for the same wind speed with different wind direction conditions. (**e**–**g**) Biological class information corresponding to response at different frequencies

Under the pressure of natural selection, spiders have developed highly specialized vibratory perception mechanisms to compensate for their degraded visual systems. Ultra-sensitive detection of mechanical vibration signals is achieved by incorporating spider webs with inherent mechanical conduction properties as vibration signal amplifiers and enhancing their sensitivity through sensilla trichoidea distributed across the appendages. The neural system of spider is capable of analyzing multimodal environmental information and accurately recognizing biophysical parameters originating from diverse vibration sources, including prey impacts, predator proximity, mate courtship behaviors, and airflow disturbances [48]. Inspired by this, a 3D bionic vibration-sensing system was developed by integrating a bionic cilia MEMS chip with a spiderweb structure using the gold wire bonding process. The architecture achieves broadband vibration response through a topologically optimized spider web structure (4 mm diameter, 25 μm wire thickness), integrated with a bionic cilia MEMS chip to create a multi-level vibration signal recognition system, as shown in Fig. 7a. When the prey or mate comes into contact with the web, the spider silk, characterized by its highly elastic protein fiber structure, effectively transmits even weak mechanical vibration waves to the sensilla trichoidea located at the basal segment of the spider’s leg. These ciliated structures transduce mechanical vibrations into bioelectric signals via nerve conduction pathways, thereby establishing a complete vibration-sensing feedback system. The 3D gold wire bionic web is designed based on bionic principles to precisely replicate the mechanical conduction properties of the spider silk. When an external object interacts with the sensing network, the gold wires transmit vibration energy via surface stress redistribution to a cilia-like structure. This structure transforms deformation into measurable electrical signal variations through the distinct flexoelectric effect, thereby enabling vibration detection akin to the suspended silk pulse diagnosis technique employed by traditional Chinese medicine practitioners, as displayed in Fig. 7b. Spiders can achieve target recognition by sensing the difference in weight (i.e., vibration amplitude) between prey and mates. 3D CGVTs featuring gold bionic spider web structures enable highly precise mass discrimination. By precisely quantifying the amplitude variations in the current signal output from the sensing unit, the vibration characteristics induced by an 80-mg target on the suspension structure can be accurately identified, as exhibited in Fig. 7c. In contrast to the indirect sensing mode, which depends on vibrational transmission via spider webs, there exists an alternative and highly efficient detection mechanism: the cilia-based direct sensing system. This system directly translates aerodynamic signals, such as flow velocity, direction, and frequency, into neuroelectrical signals through ciliated structures distributed across the body surface. As a result, it enables real-time environmental sensing without requiring any mediated transmission. As illustrated in Fig. 7d, when the airflow interacts with the cilia-like bionic structure, characteristic mechanical deformations are induced by incoming wind from various directions. Specifically, the airflow perpendicular to the axial direction of the ciliated bionic structure induces elastic compression or expansion, while the airflow parallel to the axial direction generates torsional strain. Due to the heterogeneous distribution of the surface strain field gradient, the current intensity generated in response exhibits a strong correlation, thereby enabling the precise identification of multi-directional vectors. The minimum detectable wind speed is about 0.17 m s^−1^. In addition, the vibration frequency serves as a critical criterion for distinguishing biological characteristics. Atmospheric wind-induced vibrations typically occur within the infrasound frequency band, with their frequency range predominantly concentrated between 1 and 10 Hz (Fig. 7e). In contrast to natural wind fields, the locomotor vibrations of predatory animals exhibit higher-frequency characteristics, typically ranging from 10 to 100 Hz (Fig. 7f). This range aligns with the mechanical vibration spectrum of the locomotor organs in most mammals. Notably, certain species, such as insects, are capable of producing high-frequency vibration signals ranging from 100 to 1000 Hz due to their distinct physiological adaptations (Fig. 7g). These three approaches effectively replicate the biophysical mechanisms underlying target recognition and classification through vibration in spiders, offering a novel framework for applying next-generation biomimetic vibration sensors in intelligent monitoring systems.

## Conclusions

We demonstrate a 3D CGVT fabricated via stress-induced self-assembly. The atomically thin graphene leveraged its superb mechanical properties and exceptional electrical conductivity to achieve breakthrough flexoelectric coupling. Bioinspired 3D architecture disrupts the inherent structural symmetry of graphene crystals, introducing a special flexoelectricity for electromechanical energy conversion in graphene. A vibration range of 0–1120 g and a frequency response range of 1 Hz–10 kHz were achieved, respectively. The maximum charge sensitivity of the device is up to 87.95 pC g^−1^, superior to all reported vibration transducers (or accelerometers) without mass blocks. In addition, vibration detection under 25–800 °C was achieved through the design of a silicon nitride (Si_3_N_4_) nanoprotective layer. Furthermore, direction discrimination with an accuracy of 98.31% was achieved by an omnidirectional decoupling algorithm grounded in 1DCNN. A 3D bionic vibration-sensing system was developed by integrating a bionic cilia MEMS chip with a spider web structure using a gold wire bonding process, and three different mechanisms of vibration detections and recognitions were realized. Our CGVTs could unlock a wealth of untapped potential for the advancement of vibrational transducers constructed from 2D materials, which feature superior integration, enhanced performance, and enhanced intelligence.

## Supplementary Information

Below is the link to the electronic supplementary material.

Supplementary file1 (DOCX 18826 KB)

Supplementary file2 (MP4 1193 KB)

Supplementary file3 (MP4 5295 KB)
