# Supplementary material for: Sensilla Trichoidea-Inspired, High-Temperature, and Omnidirectional Vibration Perception Based on Monolayer Graphene
Source: Nanomicro Lett. 2026 Jan 12;18:190. doi: 10.1007/s40820-025-02029-z (PMC12791082; doi:10.1007/s40820-025-02029-z)
Supplement: Supplementary file 1 — Supplementary file1 (DOCX 18826 KB) [file 40820_2025_2029_MOESM1_ESM.docx]

Supporting Information for

**Sensilla Trichoidea-Inspired, High-Temperature, and** **Omnidirectional Vibration Perception Based on Monolayer Graphene**

Yuning Li^1^^, #^, Danke Chen^1, #^, Xiaoqiu Tang^1, #^, Peizhi Yu^2, #^, Jingye Sun^1^, Xue Li^1^, Qing You^1^, Mingqiang Zhu^1^, Chang Gao^1^, Linan Li^1^, He Tian^3,^ *, Tao Deng^1,^ *

^1^ School of Electronic and Information Engineering, Beijing Jiaotong University, Beijing 100044, People’s Republic of China

^2^ Department of Precision Instrument, Tsinghua University, Beijing 100049, People’s Republic of China

^3^ School of Integrated Circuits and Beijing National Research Center for Information Science and Technology (BNRist), Tsinghua University, Beijing 100049, People’s Republic of China

*^#^*Yuning Li, Danke Chen, Xiaoqiu Tang and Peizhi Yu contributed equally to this work.

*Corresponding authors. E-mail: [tianhe88@tsinghua.edu.cn](mailto:tianhe88@tsinghua.edu.cn) (He Tian); [dengtao@bjtu.edu.cn](mailto:dengtao@bjtu.edu.cn) (Tao Deng)

**S1 Preparation Flowchart**

**
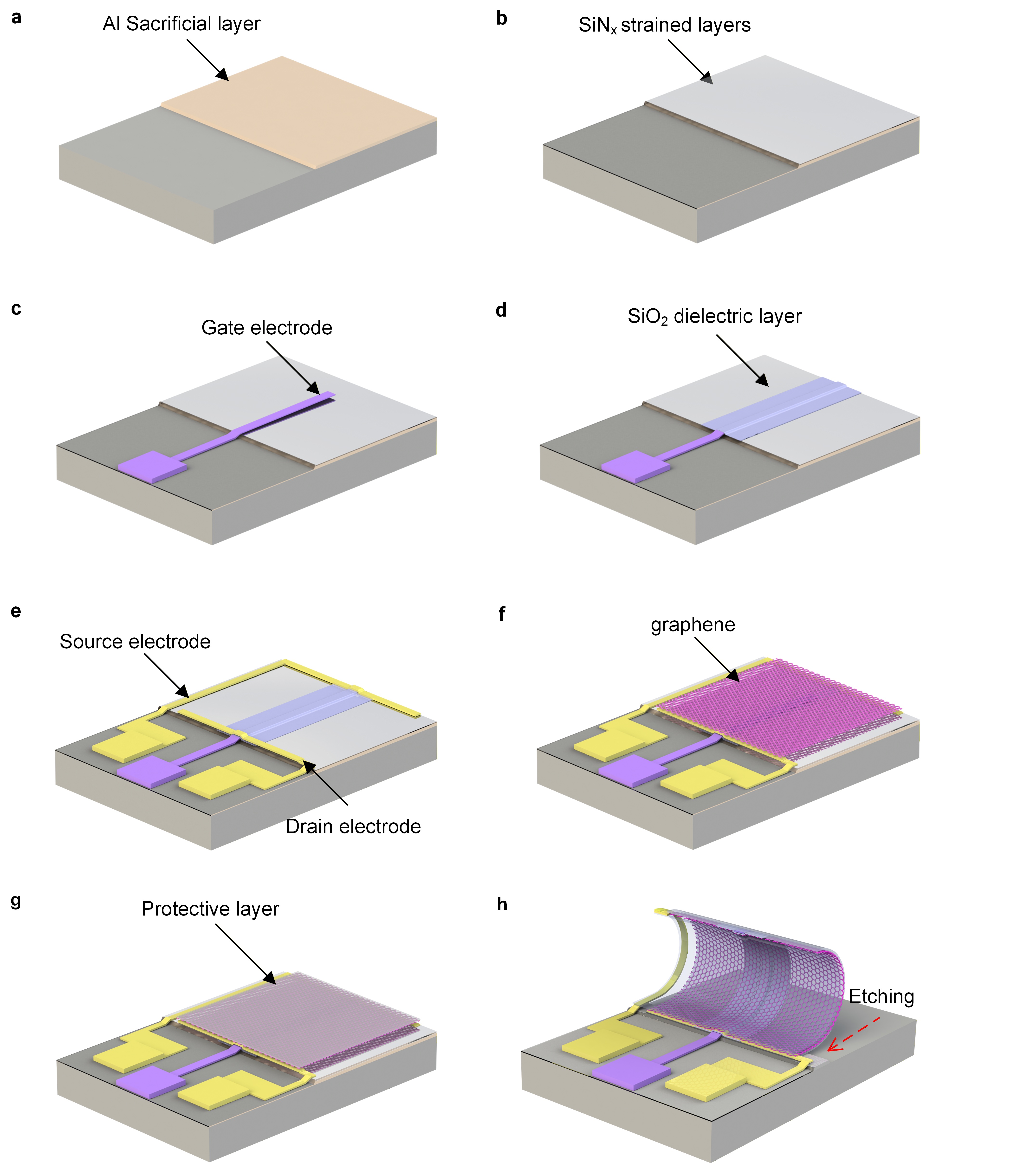
**

**Fig. S1** The fabrication process of cilia-like monolayer graphene vibration transducer (CGVT)

**S2 Yield**

We inspected 16 cycles, involving a total of 128 devices. **Figure S2a** shows the SEM and OM plots of some typical cycles. The number of successfully self-rolled-up 3D devices in each cycle is marked in the lower right corner. **Figure S2b** shows the statistical relationship between the number of cycles and the number of successfully self-rolled-up 3D devices in each cycle. In each cycle, the number of self-rolled-up 3D devices is 4,5,6,7,8, and the corresponding cycle number is 1,1,3,5,6, totaling 116. **Figure S2c** demonstrates the resistance value of the sensitive material (graphene) among 116 self-rolled-up devices. The resistance values of the devices (graphene) are mostly between 1.0 Ω and 2.0 kΩ. When the resistance value of the device is between MΩ and TΩ, it is considered a bad value, and at this point, the normal electrical properties of graphene cannot be measured. There are 101 3D devices that can work normally. Accordingly, in the red box at the lower right corner of **Fig. S2c**, the calculated yields are shown. The yield of self-rolled-up is 91%, the yield of graphene is 87%, and the overall yield of 3D devices is 79%.





**Fig. S2** **a** The SEM and optical microscope diagrams of typical cycles. The number of 3D devices that successfully self-rolled in each cycle is marked in the lower right corner. **b** The relationship between the number of cycles and the number of self-rolled-up 3D devices in each cycle. **c** The resistance value of 116 3D devices. The yield rates are displayed in the red box at the lower right corner.

**S3 Device morphology**

**
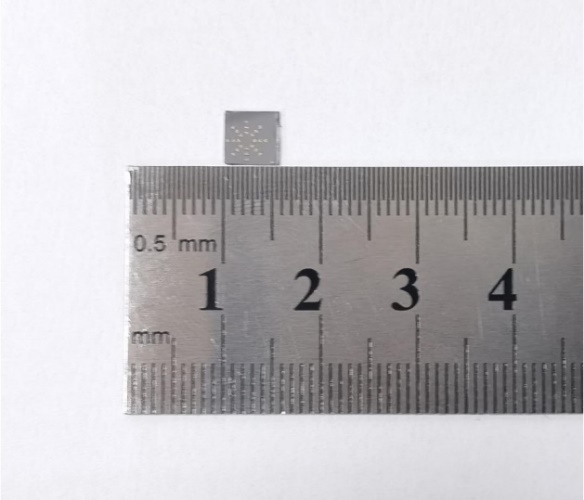
**

**Fig. S3** The optical image of a monolithic integrated petal-like CGVT array with a steel ruler

**
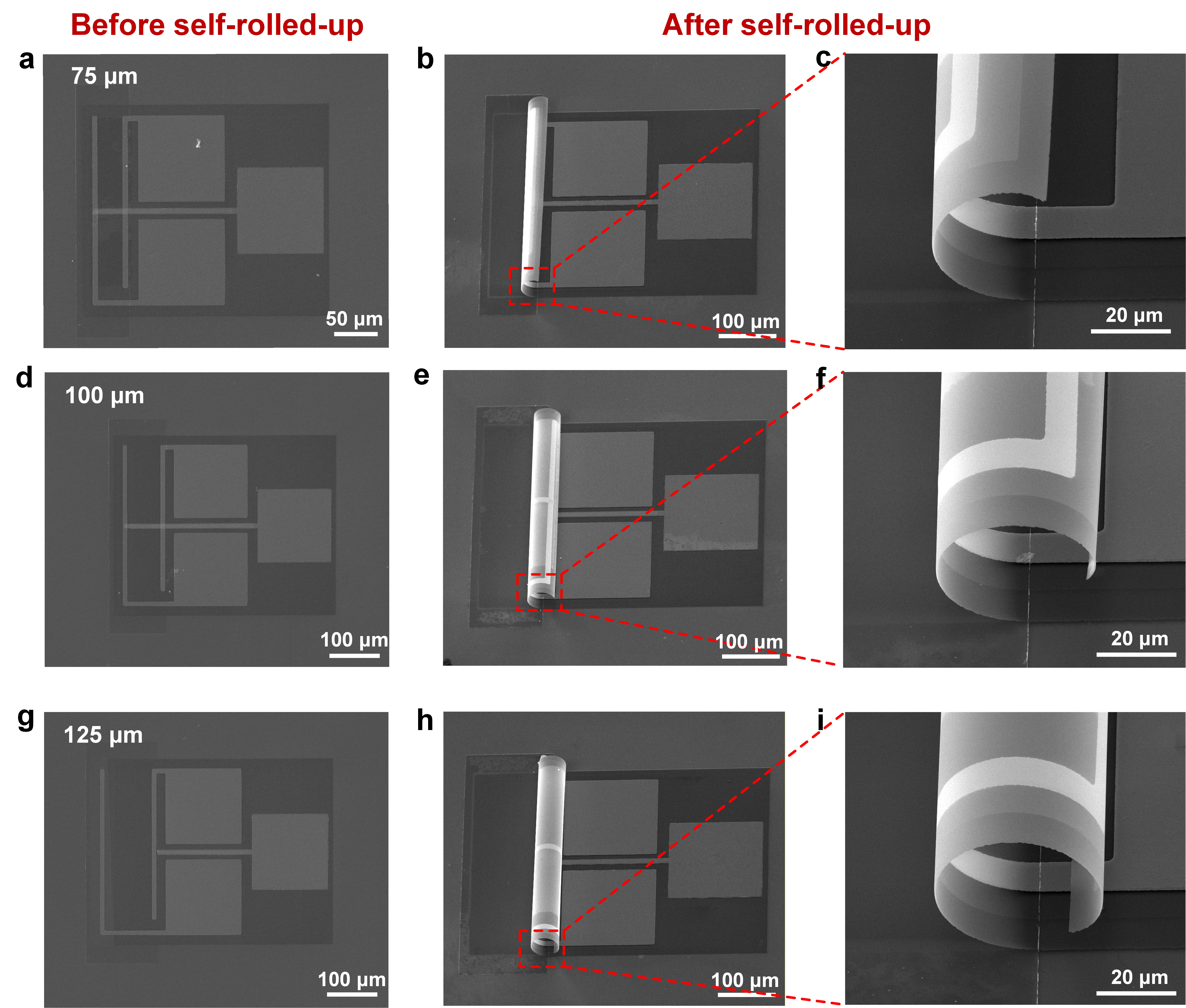
**

**Fig. S4** The SEM images of the CGVTs before and after self-rolled-up with varying sizes

**S4 Electrical connection diagram for vibration testing**

The response of the CGVT can be monitored through two distinct approaches: one involves measuring the short-circuit current by configuring the B2911 precision power module in voltage mode with the voltage set to zero; the other entails measuring the open-circuit voltage by operating the B2911 in current mode with the current set to zero (as illustrated in the schematic monitoring circuit **Fig. S5**). The short-circuit current measurement was uniformly chosen in the manuscript to assess the CGVT’s vibrational response.


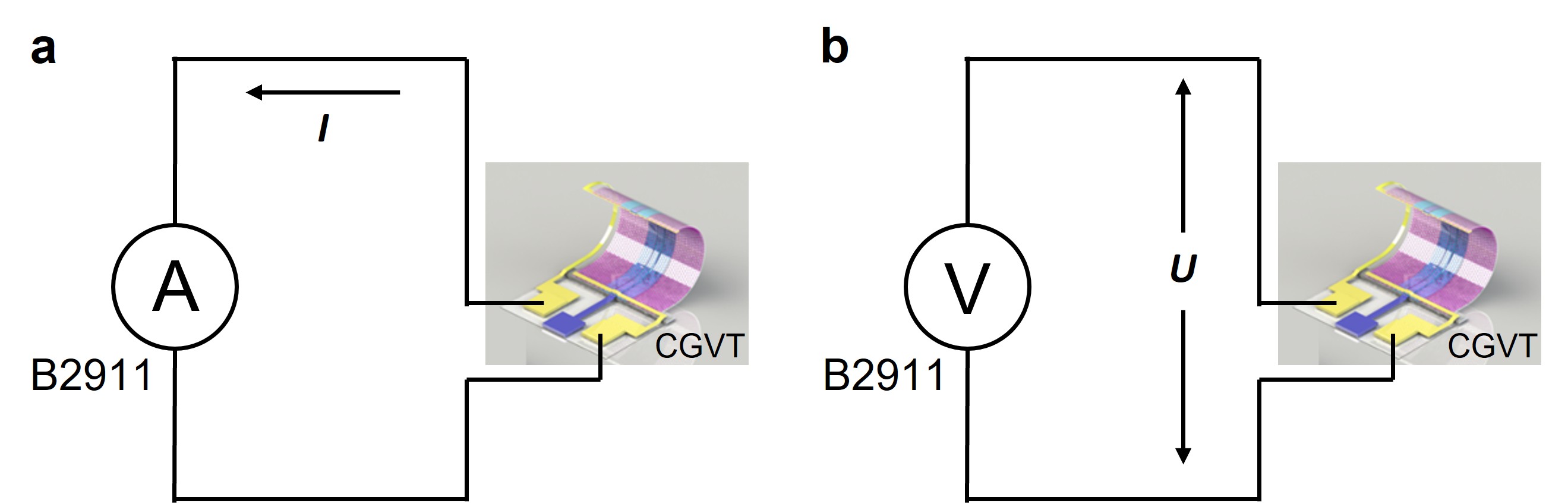


**Fig. S5** Monitoring circuit diagrams of the CGVT under **a** Open-circuit voltage and **b** short-circuit current conditions

This study employed a calibration path involving direct comparison with a reference standard accelerometer (HBK, 8305) that had been calibrated using an absolute method. The tested vibration transducer was installed coaxially with the reference accelerometer mounted inside the fixture. When both the reference standard accelerometer and the transducer under test (CGVT) respond to the same vibration parameter, the sensitivity *S*_2_ of the CGVT should be calculated as follows:

 (S1)

*S*_1_ and *X*_1_ represent the sensitivity of the standard sensor and its output value, respectively. *S*_2_ and *X*_2_ denote the sensitivity magnitude and output value of the transducer under test. A schematic diagram of the complete sensitivity test system is shown in **Fig. S6**. This system enables direct output of the sensitivity (unit: pC/g) of the transducer under test via a computer equipped with vibration transducer calibration software. Meanwhile, the current of the CGVT under zero-bias conditions was monitored in real time using a precision source/measure unit (B2911A). The corresponding monitoring circuit is presented in Fig. 4a. Furthermore, the performance of piezoelectric accelerometers is typically quantified by charge sensitivity (pC/g) and voltage sensitivity (mV/g). In this work, we employ charge sensitivity to characterize the CGVT’s performance metrics. Regarding the current sensitivity (mA/g) mentioned, the current-output accelerometer typically requires an external current-to-voltage conversion integrated circuit for signal output.


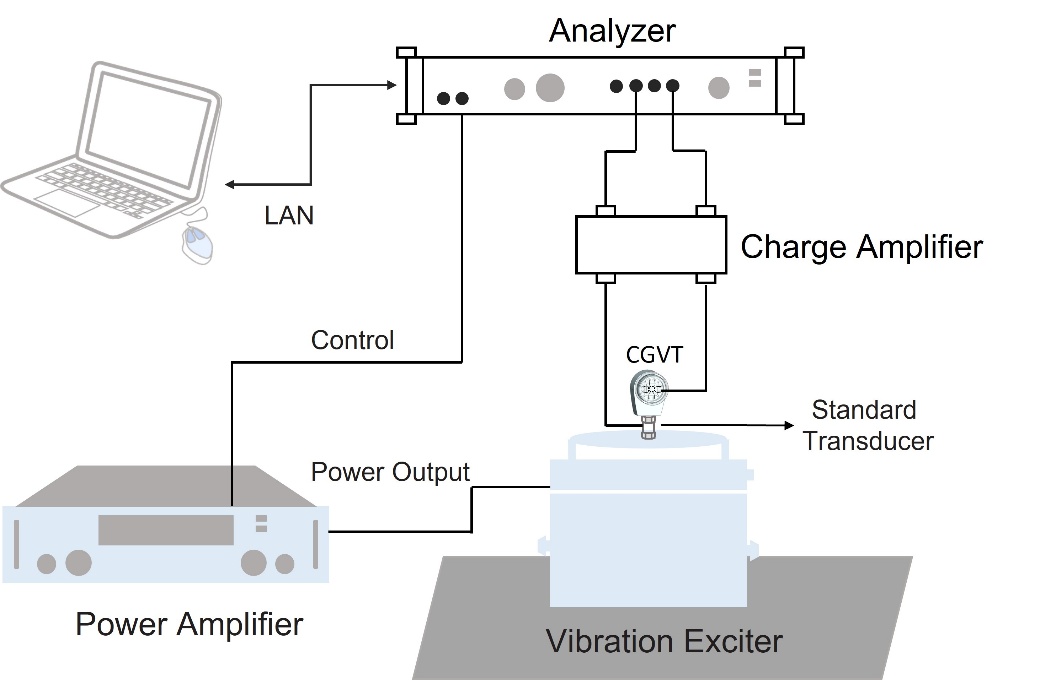


**Fig. S6** Sensitivity comparison method calibration system

**S5 Noise analysis**

**Fig. S7** Time-domain current noise signal of the device

**S6 Frequency response**

**
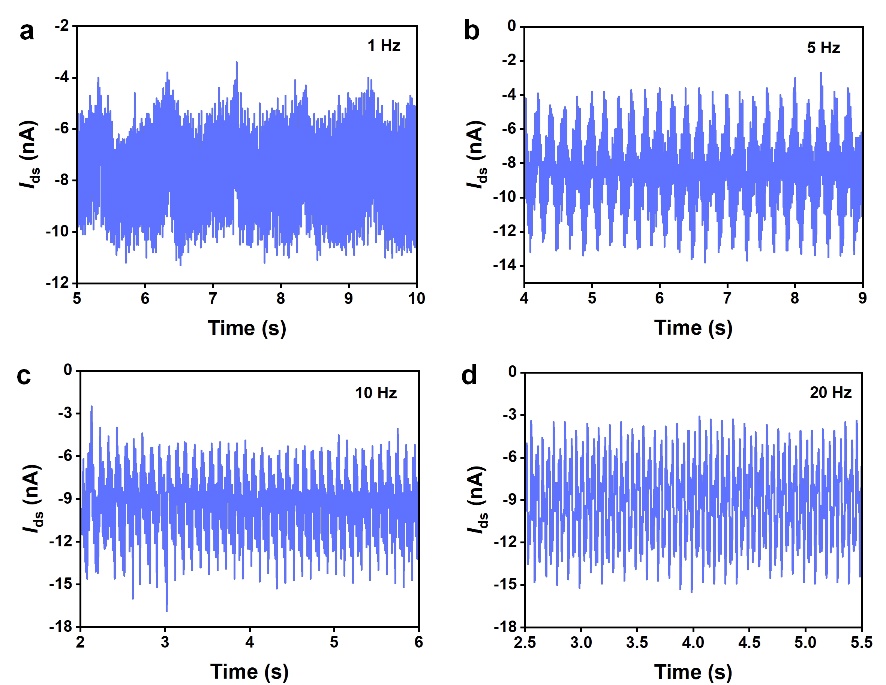
**

**Fig. S8** Time-domain current response signal of the device under 1~20 Hz vibration

**
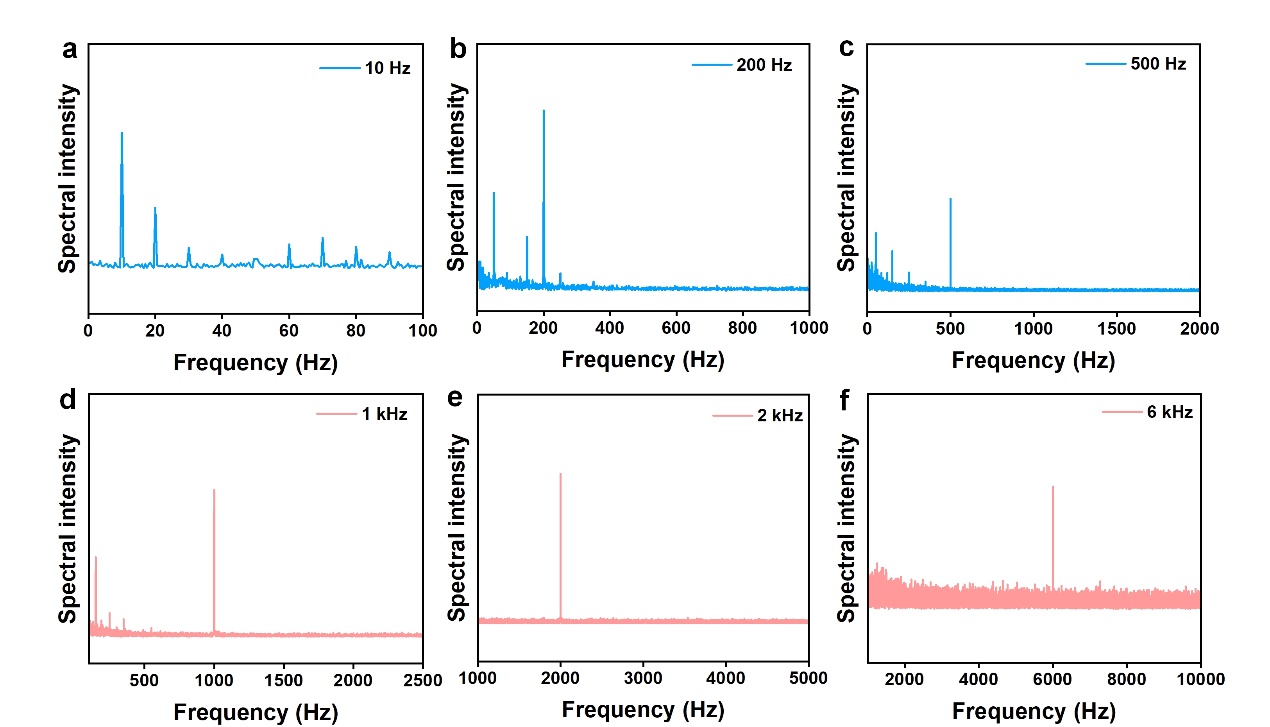
**

**Fig. S9** Frequency-domain response of the CGVT under different vibration excitation


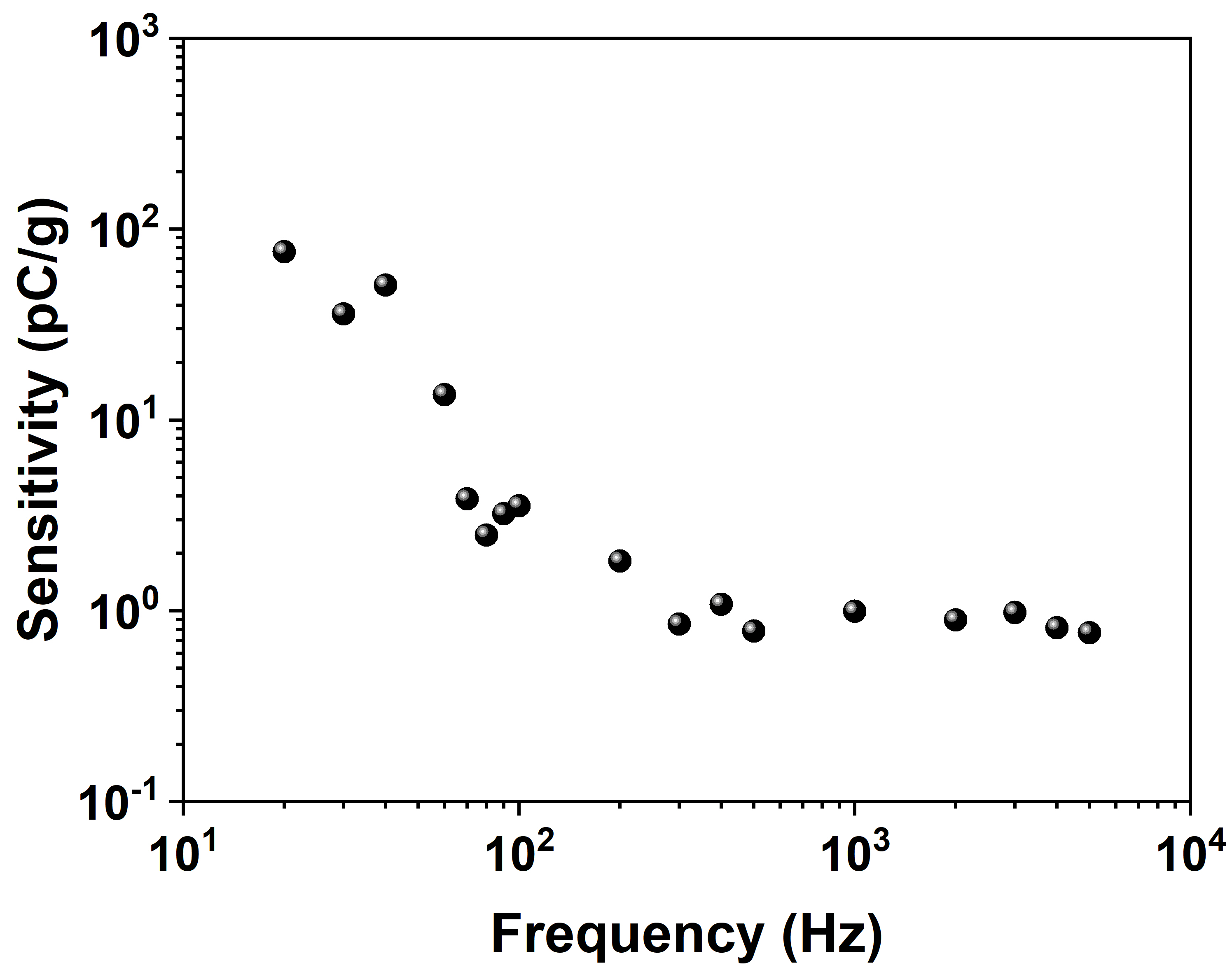


**Fig. S10** Charge sensitivity as a function of frequency

**S7 Stimulation of vibration transducer (devices/array) in COMSOL**

① General setup

The finite element analysis simulations are performed with the COMSOL Multiphysics software. In the calculation process, electric field and stress field are coupled together for calculation, and they will interact with each other. In the simulation, three materials are considered: the bottom layer is the substrate, above which lies a 200 nm thick layer of silicon nitride, and on top of that is an ultra-thin layer of graphene (the original thickness is 0.335 nm, but it is slightly thickened in the actual model to avoid convergence issues due to extremely thin materials in simulations). Part of the silicon nitride and graphene layers are curled with a radius of 22.5 μm. In the simulation, a sinusoidal external force is applied along the length direction of the substrate, causing periodic displacement in that direction to match the actual experimental conditions. This utilizes inertia to drive the curled silicon nitride and graphene layers above, inducing bending deformation, and subsequently enabling the calculation of the polarization electric field distribution. The following figure shows the basic model:


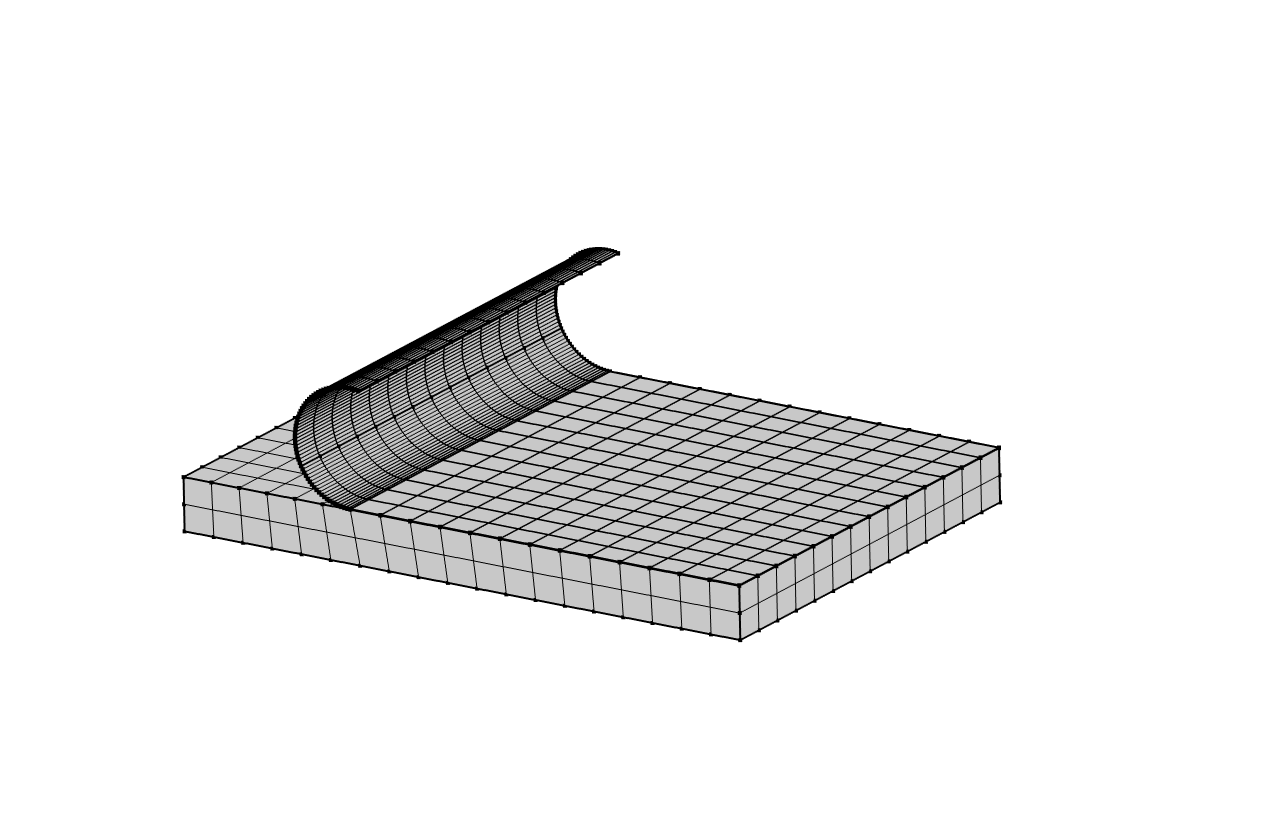


**Fig. S11** Geometric modeling and meshing of the device

② Material parameter

| Materials | Young's modulus [Gpa] | Poisson's ratio |
| --- | --- | --- |
| Graphene | 1050 | 0.186 |
| Silicon Nitride | 300 | 0.24 |
| Silicon | 170 | 0.28 |

③ The basic equations

⮚ Solid mechanics equations

For mechanical simulation, the displacement of materials is calculated according to the above equations, and the kinematic stress strain constitutive relation is given by Hooke’s Law:

 (S2)

 (S3)

 (S4)

where *σ*_x_, *σ*_y_, *σ*_z_ means the normal stresses for x, y, z directions. *ϵ*_x_, *ϵ*_y_, *ϵ*_z_ means the normal strains, *E* is the Young’s modulus, *ν* is the Poisson’s ratio. The vibration loading is achieved by applying a periodic surface load to the backside of the substrate, which is consistent with the method used to apply force during the experimental vibration of the device. We mainly investigated the stress distribution in the resting and vibrating states of the device under the influence of gravity. The Von Mises stress is expressed as,

Von Mises stress = $\sqrt{\frac{\left(\sigma_{1}-\sigma_{2}\right)^{2}\text{+}\left(\sigma_{2}-\sigma_{3}\right)^{2}\text{+}\left(\sigma_{3}-\sigma_{1}\right)^{2}}{2}}$ (S5)

where *σ*_1_, *σ*_2_, *σ*_3_ are the first, second and third principal stresses, respectively.

⮚ Constitutive Equations (Ferroelectric Material)

The stress (σ) and electric displacement (D) are coupled via ferroelectric and elastic effects:

$\mathbf{\sigma}=\mathbf{c}^{E}:\mathbf{\varepsilon}-\mathbf{e}^{T}\cdot\mathbf{E}$ (S6)

$\mathbf{D}=\mathbf{e}:\mathbf{\varepsilon}+\mathbf{\kappa}^{\varepsilon}\cdot\mathbf{E}+\mathbf{P}_{r}$ (S7)

where c^E^ means the Elastic stiffness tensor at constant electric field, e means the piezoelectric stress coefficient tensor, e^T^ is the transpose of the piezoelectric tensor, E is the electric field vector, κ^ε^ is the dielectric permittivity tensor at constant strain, P_r_ is the Remnant polarization vector.

⮚ Gauss’s Law for Electric Displacement

The polarization charge density (p) is derived from:

$\rho_{p}=-\nabla\cdot\mathbf{P}$ (S8)

where *ρ*_p_ means the free charge density, P means the total polarization. By solving the above equations simultaneously, the distributions of electric field, ferroelectric potential, and stress field can be obtained.

**S8 Performance degradation at high temperature**

**Figure S12a** shows the optical microscope diagrams of the devices after treatment at different temperatures, and the corresponding Raman spectra are presented in **Fig. S12b**. Unlike the Raman spectra of graphene without protective layers reported in the literature [S1], the intensity of D band located at the 1349.99 cm^-1^ remains basically unchanged with the increase of temperature below 600℃. The negligible D band indicate that there are basically no structural defects in the graphene layers of each sample [S2]. Therefore, the Si_3_N_4_ protective layers achieve effective protection. After being treated at 800℃, the strength of the D band significantly increased, but at this time, monolayer graphene could still function normally. However, some 3D devices have their stress layers relaxed and expanded into 2D planar devices due to high temperature. When in contact with the substrate, due to the mismatch of the thermal expansion coefficients between the material and the substrate, the material shows bubbles and cracks. At this point, its Raman spectrum is displayed at the top of **Fig. S12b**, indicating that there is no longer graphene present. Graphene has failed due to oxidation when exposed to a high-temperature oxygen-rich environment. In addition, by combining **Fig. S12a** and the corresponding Raman spectra of **Fig. S12b**, it can be seen that after the device was subjected to a high temperature of 800 ℃ and vibration, the device still maintains mechanical integrity and can operate normally. However, its radius of curvature decreases, which is consistent with the reduction in the diameter of 3D semicircular cantilever devices after thermal annealing treatment in the literature [S3].





**Fig. S12** **a** The optical microscope diagrams and **b** Raman spectra of 3D graphene devices after treatment at different temperatures.

**S9 Benchmark**

**Table S1** Benchmark of vibration sensors

| **Description** | **With/Without Mass Block** | **Device Dimensions** | **Measuring Range** | **Frequency Range** | **Sensitivity** | **Noise Density** | **Minimum Detectable Level** | **Temperature Range** | **Lifetime** | **References** |
| --- | --- | --- | --- | --- | --- | --- | --- | --- | --- | --- |
| AlN | with | / | 0-10 g | 1 Hz-5 kHz | 1.49 mV/g | / | / | / | / | [S4] |
| PZT | with | 5 × 5 × 5 mm³ | 0-4 g | 1 Hz-500 Hz | 0.75 pC/g | / | / | / | / | [S5] |
| PVDF | with | 15 mm² | 0.5 g-6 g | 58.6 Hz | 21.82 pC/g | 6.07 μg/√Hz | 257.7 μg | / | / | [S6] |
| PVDF | with | 225 mm² | 0.5 g-10 g | 160 Hz | 29.45 pC/g | 1.40 μg/√Hz | / | / | / | [S7] |
| AlN | with | 45.8 mm² | 0.001 g-10 g | 1.1 kHz | 5.2 pC/g | 670 ng/√Hz | / | / | / | [S8] |
| AlN | with | 2×2 mm² | / | 600 Hz-10 kHz | 3.8-3.9 fC·s²/m | 7.7 mm/s²/√Hz | / | / | / | [S9] |
| AlN | with | 2×2 mm² | / | 250 Hz-8 kHz | 10.7 fC·s²/m | 0.2 mm/s²/√Hz | / | / | / | [S10] |
| PVDF | with | / | 0-9 g | 100 Hz-2500 Hz | 134.59 mV/g | / | / | 20~25°C | / | [S11] |
| PZT | with | 20.97 mm³ | / | 10-200 Hz | 22.74 pC/g | 5.6 μg/√Hz | 279 μg | / | / | [S12] |
| AlN | with | / | / | 10-900 Hz | 1.3 mV/g | 36.3 nV/√Hz | / | / | / | [S13] |
| YCOB | with | / | 0.2 g-0.8 g | 200-800 Hz | 1.9 ± 0.4 pC/g | / | / | RT~1000°C | 3 h@1000°C | [S14] |
| YCOB | with | / | 1 g-5 g | 80-1000 Hz | 5.7 pC/g | / | / | RT~1000°C | 4 h@1000°C | [S15] |
| AlN | with | / | 1 g-5 g | ≤ 600 Hz | 9.2 pC/g | / | / | RT~1000°C | 10 h@1000°C | [S16] |
| YCOB | with | / | / | 100-600 Hz | 2.4 pC/g | / | / | 20~900°C | 3 h@900°C | [S17] |
| PZT | with | / | 1 g-10 g | ≤ 10 kHz | 79.4-296.8 pC/g | / | / | / | / | [S18] |
| PZT | with | / |  | 60 Hz-1.5 kHz | 3.4-50 pC/g | 1.7 μg/√Hz | / | / | / | [S19] |
| PZT | with | 10×10 mm² | 0.5 g-45 g | 0.1-4 kHz | 0.23 pC/g | / | 1 g | / | / | [S20] |
| ScAlN /AlN | with | / | 0.2 g-2 g | 50-440 Hz | 7.95 mV/g | 92.2 nV/√Hz | 1 mg | RT | / | [S21] |
| BST | with | / | 0.2 g-0.8 g | 30-100 Hz | 2.8 pC/g | / | / | RT | / | [S22] |
| BTS | with | / | 0-4 g | 100-600 Hz | 2.61-2.62 pC/g | / | / | 25~650°C | 1 h@650°C | [S23] |
| ZnO | with | / | / | 1200 Hz | 1.96 mV/g | 0.267 mg/√Hz | / | / | / | [S24] |
| LGS | with | 25×30×6 mm³ | ±1 g | 80-850 Hz | 3.29 pC/g | / | 10 mg | 25~800°C | 6 h@800°C | [S25] |
| AlN/ScAlN | with | / | 0.2 g-2.2g | 3 Hz-1 kHz | 3.35 pC/g | / | / | / | / | [S26] |
| ZnO | with | / | 0-30 g | 2 kHz-20 kHz | 1.69 mV/g | / | / | / | / | [S27] |
| PLZT | with | / | 1 g-10 g | 2 kHz-20 kHz | / | / | / | / | / | [S28] |
| PZT | with | / | 0.001 g-2 g | 3 kHz | 8.12 mV/g | 5.8 mg/√Hz | 5.8 mg | / | / | [S29] |
| PZT | with | / | 0-10 g | 10 Hz-1 kHz | 2.21 mV/g | / | / | / | / | [S30] |
| PZT | with | 6 × 6 mm² | / | 3.7-35.3 kHz | 0.77-7.6 pC/g | / | 30 µg | / | / | [S31] |
| ZnO | with | 15 × 15 mm² | 0-15 g | 0-17 Hz | 16.1-16.3 mV/g | / | / | / | / | [S32] |
| LiNbO_3_ | with | 10 × 10 mm² | 5 g-20 g | 20-2400 Hz | 5.2-10.3 pC/g | / | / | -40°C~+70 °C | / | [S33] |
| LGT | with | 34×30×20 mm³ | / | 100 Hz-2000 Hz | 3.3 pC/g | / | / | 20°C~350°C | / | [S34] |
| BSO | with | / | 1 g-6 g | 160 Hz | 3.89 pC/g | / | / | 25~650°C | / | [S35] |
| AlN | with | 2.3×2.3 mm² | 0.2 g-3 g | 10 kHz | 440 μV/g | 200 μg/√Hz |  | / | / | [S36] |
| AlN | with | / | ±5 g | 24.66 kHz | 346 ppm/g | / | / | -40°C~+85°C | / | [S37] |
| AlN | with | 464×650 μm² | ±5 g | 16.1 kHz | 68.9 ppm/g | / | / | 0~50°C | / | [S38] |
| AlN | with | 868×833 μm² | 0.1 g-2 g | 200 Hz-1.5 kHz | 1.553 mV/g | 841 nV/√Hz | 2 mg | / | / | [S39] |
| BiFeO₃/BaTiO₃ | with | / | / | / | 40 pC/g | / | / | RT~400°C | / | [S40] |
| ScAlN | with | 2.2×2.2 mm² | 0.1 g–2 g | 56 Hz-2360 Hz | 2.448 mV/g | 85.6 nV/√Hz | 1 mg | / | / | [S41] |
| LGT | with | / | 0-40 g | 10 Hz-2500 Hz | 1.046 pC/g | / | / | 20~700°C | / | [S42] |
| LiNbO₃ | with | 11 × 11 mm² | 5 g-20 g | 20 Hz-2400 Hz | 10.336 pC/g | / | / | / | / | [S43] |
| PVDF | without | / | 0-50000 g | / | 0.177 ± 0.03 pC/g | / | / | / | / | [S44] |
| Al/PDMS | with | 20 × 20 × 8 mm³ | 0-1000 g | / | 50.89 mV/g | / | 0.69 g | -40°C~+50 °C | / | [S45] |
| BTO/PEGDA/VMM | with | 12 × 4.5 mm² | 0.1 g-0.5 g | ≤17 Hz | 210.9 mV/g | / | / | / | / | [S46] |
| **Our work** | **without** | **0.373 ×0.3 mm²** | **0-1120 g** | **1 Hz-10 kHz** | **87.95 pC/g** |  | **0.53 g** | **25-800 °C** | **4 h@25-800 °C** |  |

**S10 1DCNN-based direction decoupling**

(1) Loss Function and Accuracy

The cross-entropy loss function is used to evaluate the error of the predicted output and the true labels. Suppose p and q is the true distribution and the predicted distribution, respectively. The loss function (*L*) is expressed as follows:

$L=-\sum_{\text{i}=1}^{\text{k}} p_{\text{i}}\log\left(q_{\text{i}}\right)$ (S9)

For the classification task, accuracy is an important indicator to evaluate the proportion of predictions that are correct. If it is a binary task, accuracy formula is presented in Equation (S10):

$Accuracy=\frac{TP+TN}{TP+TN+FP+FN}$ (S10)

where TP, FP, TN, and FN refer to the number of true positive, false positive, true negative, and false negative predictions, respectively. The multi-classification task can be regarded as multiple binary classification tasks.

(2) Parameter Settings and Experimental Environment

In our 1DCNN model, the kernel size, stride and padding of each convolutional layer are 3, 1, 1, respectively, and the kernel size of each max pooling layer is 2. The neural network was evaluated on vibration dataset containing different amplitudes, frequencies and noises. The dataset contains four different amplitudes datasets, each of which includes data collected from seven vibration directions ([001], [010], [011], [100], [101], [110], [111]), each with distinct frequencies and noises. Each vibration direction contains 760 samples, and each sample is the time-domain current value of a circle of eight sensors vibration for 5 s collected. The time-domain current values were recorded at sampling points of 5 ms. The dataset is randomly divided into a training set and a test set in an 8:2 ratio, with a batch size of 128. Furthermore, the loss function is cross-entropy loss, and the optimizer is Adam. The initial learning rate is set at 0.001, which is then adjusted by the StepLR learning rate scheduler, and the dropout rate is set at 0.5. To prevent overfitting, weight decay is introduced into the model. At the same time, data augmentation techniques including random time distortion, amplitude drift and data loss are adopted to enhance the robustness and generalization ability of the model. To further verify the model’s performance, three types of verification experiments were conducted: First, simulating three disturbance scenarios of time axis stretching/compression of time series data, 10% data loss, and amplitude drift (±20%), to test the model’s performance under harsh data conditions. Second, the data augmentation module for ablation experiment was adopted. By constructing a control model that did not apply data augmentation technology, and retraining it with the same dataset partitioning and training parameters, the robustness indicators of the two groups of models are compared to verify the improvement effect of data augmentation on model performance. Third, leave-one dataset was retained for validation. Four datasets of different magnitudes are alternately used as the test set, and the rest are used as the training set. For each dataset, 10% of the samples are selected to participate in training and testing. Through multiple rounds of validation, the cross-dataset adaptability of the model was evaluated. Our model was implemented in Python 3.12 with Pytorch frameworks on Windows 11 operating system with Intel Core i9-14900HX CPU and 32 GB RAM.

The validation of the 1DCNN model

To further verify the model’s performance, three types of validation experiments were conducted: First, simulating three disturbance scenarios of random time distortion (stretching/compressing the timeline of time series data), 10% data loss, and amplitude drift (±20%), to test the model’s performance under harsh data conditions. At the same time, the data augmentation module for ablation experiment was adopted. By constructing a control model that did not apply data augmentation technology, and retraining it with the same dataset partitioning and training parameters, the robustness indicators of the two groups of models are compared to verify the improvement effect of data augmentation on model performance. As shown in Figure S13, in these three perturbation scenarios, the application of data augmentation techniques can significantly improve the accuracies of the model output. It indicates the necessity of applying data augmentation and the good robustness of the model.


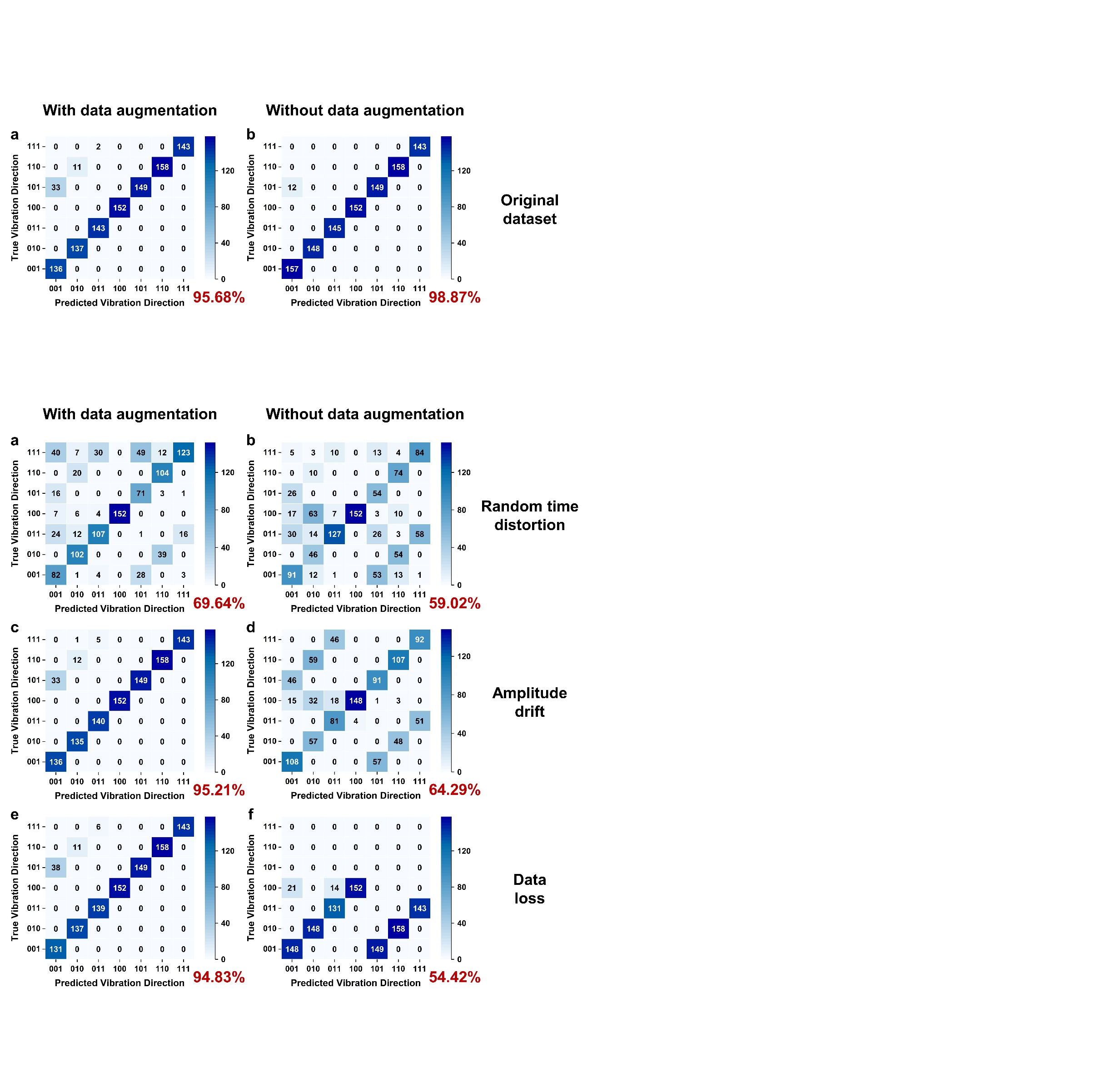


**Fig. S13** The confusion matrix output by the model **a** with and **b** without data augmentation at random time distortion. The confusion matrix output by the model **c** with and **d** without data augmentation at amplitude drift. The confusion matrix output by the model **e** with and **f** without data augmentation at data loss. The corresponding accuracies are marked in the lower right corner.

Third, leave-one dataset was retained for validation. Four datasets of different magnitudes are alternately used as the test set, and the rest are used as the training set. For each dataset, due to the large sample size, 10% of the samples are selected to participate in training and testing. Through multiple rounds of validation, the cross-dataset adaptability of the model was evaluated. The results of the four-rounds validation are shown in Figure S14, and the validation accuracy is ultimately averaged at 96.96%. High accuracy indicates that the model can learn and fit the patterns of the data very well and has strong generalization ability.

**
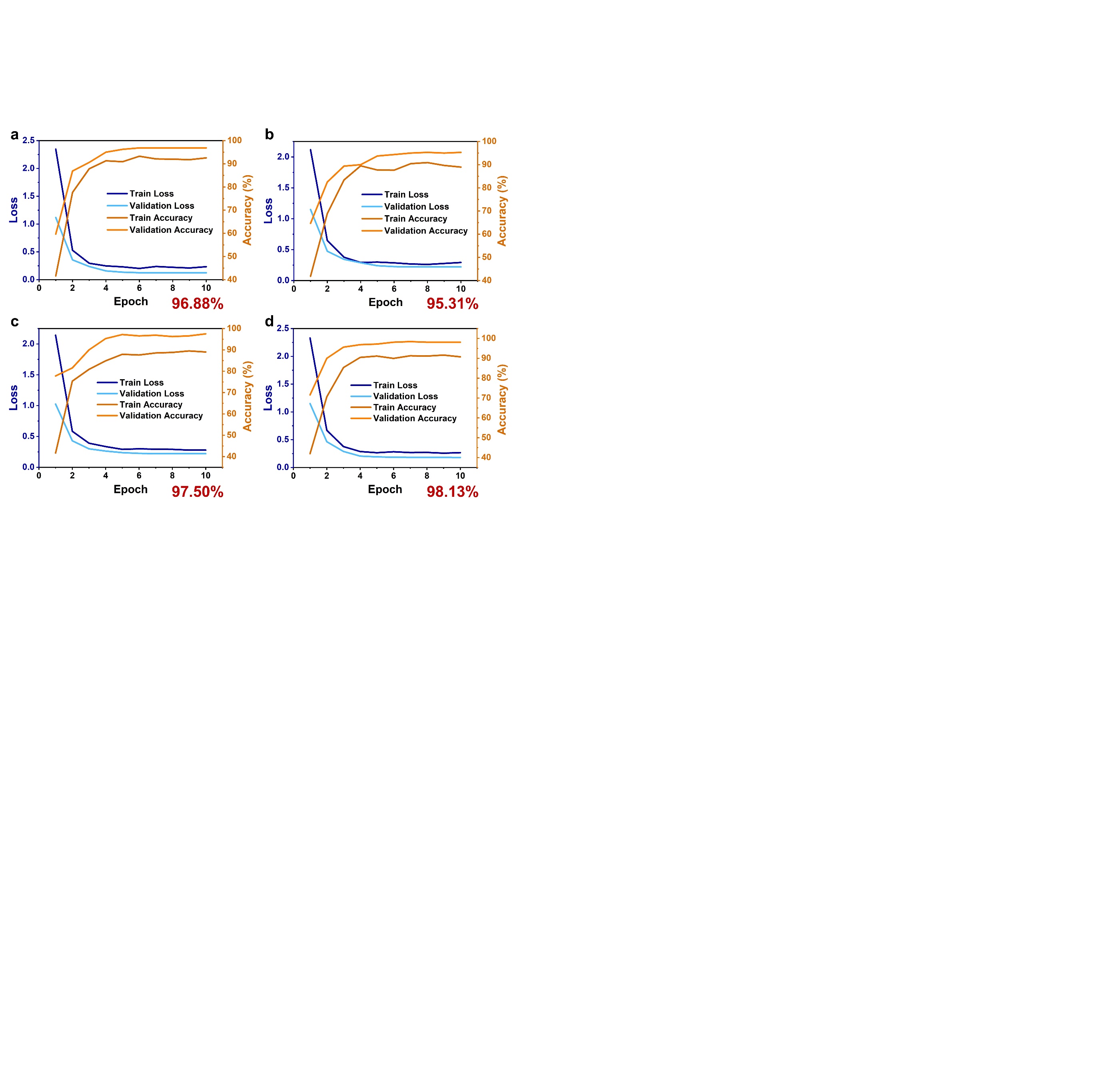
**

**Fig. S14** The loss and accuracy results of the four-rounds validation of the model with leave-one-amplitude test. The final validation accuracy of each round is marked in the lower right corner

**Supplementary References**

1. H.Y. Nan, Z.H. Ni, J. Wang, Z. Zafar, Z.X. Shi et al., The thermal stability of graphene in air investigated by Raman spectroscopy. J. Raman Spectroscopy **44**(7), 1018-1021 (2013). <https://doi.org/10.1002/jrs.4312>
2. M. Kalisz, M. Grobelny, M. Świniarskib, P. Firek, Comparison of the structural and corrosion properties of the graphene/SiN(200) coating system deposited on titanium alloy surfaces covered with SiN transition layers. Surface and Coatings Technology. **299**, 65-70 (2016). <https://doi.org/10.1016/j.surfcoat.2016.04.064>
3. A. Khandelwal, Z. Ren, S. Namiki, Z. Yang, N. Choudhary et al., Self-rolled-up aluminum nitride-based 3d architectures enabled by record-high differential stress. ACS Appl. Mater. Interfaces. **14**(25), 29014-29024 (2022). <https://doi.org/10.1021/acsami.2c06637>
4. Z.-H. Chen, C.-Y. Li, S.-Y. Chu, C.-C. Tsai, Y.-H. Wang et al., The design of aluminum nitride-based lead-free piezoelectric MEMS accelerometer system. IEEE Trans. Electron Devices **67**(10), 4399–4404 (2020). <https://doi.org/10.1109/ted.2020.3019230>
5. A. Dabrowski, K. Elkjaer, L. Borregaard, T. Zawada, L. Golonka, LTCC/PZT accelerometer in SMD package. Microelectron. Int. **31**(3), 186–192 (2014). <https://doi.org/10.1108/mi-10-2013-0052>
6. C. Ge, E. Cretu, A polymeric piezoelectric MEMS accelerometer with high sensitivity, low noise density, and an innovative manufacturing approach. Microsyst. Nanoeng. **9**, 151 (2023). <https://doi.org/10.1038/s41378-023-00628-7>
7. C. Ge, E. Cretu, Polymeric piezoelectric accelerometers with high sensitivity, broad bandwidth, and low noise density for organic electronics and wearable microsystems. Microsyst. Nanoeng. **10**, 61 (2024). <https://doi.org/10.1038/s41378-024-00704-6>
8. F. Gerfers, M. Kohlstadt, L.-P. Wang, H. Bar, T. Northemann et al., An ultra low-noise vibration monitoring system. 2007 IEEE Sensors. October 28-31, 2007. Atlanta, GA, USA. IEEE, (2007).: 880-883. <https://doi.org/10.1109/icsens.2007.4388542>
9. A.L. Gesing, F.D.P. Alves, S. Paul, J.A. Cordioli, On the design of a MEMS piezoelectric accelerometer coupled to the middle ear as an implantable sensor for hearing devices. Sci. Rep. **8**(1), 3920 (2018). <https://doi.org/10.1038/s41598-018-22219-7>
10. A.L. Gesing, Z.N. Masson, D.C. Arellano, F. Alves, S. Paul et al., Middle ear ossicular chain vibration detection by means of an optimized MEMS piezoelectric accelerometer. IEEE Sens. J. **19**(6), 2079–2086 (2019). <https://doi.org/10.1109/JSEN.2018.2886043>
11. Y. Gong, H. Zhao, Y. Huang, X. Jin, Design and experimental study of acceleration sensor based on PVDF piezoelectric film. J. Comput. Meth. Sci. Eng. **21**(1), 31–40 (2021). <https://doi.org/10.3233/jcm-204337>
12. X. Gong, Y.-C. Kuo, G. Zhou, W.-J. Wu, W.-H. Liao, An aerosol deposition based MEMS piezoelectric accelerometer for low noise measurement. Microsyst. Nanoeng. **9**, 23 (2023). <https://doi.org/10.1038/s41378-023-00484-5>
13. A.E. Hake, C. Zhao, W.-K. Sung, K. Grosh, Design and experimental assessment of low-noise piezoelectric microelectromechanical systems vibration sensors. IEEE Sens. J. **21**(16), 17703–17711 (2021). <https://doi.org/10.1109/JSEN.2021.3085825>
14. K. Kim, X. Jiang, S. Zhang, A high temperature piezoelectric sensor for structure health monitoring. Nondestruct. Charact. Compos. Mater. Aerosp. Eng. Civ. Infrastruct. Homel. Secur. 2011 **7983**, 79832V (2011). <https://doi.org/10.1117/12.880163>
15. K. Kim, S. Zhang, W. Huang, F. Yu, X. Jiang, YCa_4_O(BO3)_3_ (YCOB) high temperature vibration sensor. J. Appl. Phys. **109**(12), 126103 (2011). <https://doi.org/10.1063/1.3598115>
16. H. Kim, S. Kerrigan, M. Bourham, X. Jiang, AlN single crystal accelerometer for nuclear power plants. IEEE Trans. Ind. Electron. **68**(6), 5346–5354 (2021). <https://doi.org/10.1109/TIE.2020.2992002>
17. M.I. Lapsley, E.F. Alberta, R. Sahul, W. Hackenberger, X. Jiang et al., Piezoelectric structural sensor technology for extreme environments. Addit. Conf. Device Packag. Hitec Hiten CICMT **2010**, 355–358 (2010). <https://doi.org/10.4071/hitec-ealberta-tha21>
18. M.-K. Lee, S.-H. Han, J.-J. Park, G.-J. Lee, A theoretical and empirical investigation of design characteristics in a Pb(Zr, Ti)O_3_-based piezoelectric accelerometer. Sensors **20**(12), 3545 (2020). <https://doi.org/10.3390/s20123545>
19. N.N. Hewa-Kasakarage, D. Kim, M.L. Kuntzman, N.A. Hall, Micromachined piezoelectric accelerometers *via* epitaxial silicon cantilevers and bulk silicon proof masses. J. Microelectromech. Syst. **22**(6), 1438–1446 (2013). <https://doi.org/10.1109/JMEMS.2013.2262581>
20. C.C. Hindrichsen, J. Larsen, E.V. Thomsen, K. Hansen, R. Lou-Møller, Circular piezoelectric accelerometer for high band width application. SENSORS, 2009 IEEE., 475–478. IEEE (2010).
21. B. Hu, Y. Liu, B. Lin, G. Wu, W. Liu et al., A novel trapezoidal ScAlN/AlN-based MEMS piezoelectric accelerometer. IEEE Sens. J. **21**(19), 21277–21284 (2021). <https://doi.org/10.1109/JSEN.2021.3101219>
22. W.B. Huang, S.R. Kwon, F.G. Yuan, S.J. Zhang, X.N. Jiang, Asme. A FLEXOELECTRIC MICRO-ACCELEROMETER. ASME International Mechanical Engineering Congress and Exposition. 597-603 (2012).
23. C. Jiang, X. Liu, F. Yu, S. Zhang, H. Fang et al., High-temperature vibration sensor based on Ba_2_TiSi_2_O_8_ piezoelectric crystal with ultra-stable sensing performance up to 650 ℃. IEEE Trans. Ind. Electron. **68**(12), 12850–12859 (2021). <https://doi.org/10.1109/TIE.2020.3044792>
24. C.-Y. Li, Y.-H. Chen, Z.-Y. Wei, Y.-C. Ho, S.-Y. Chu et al., Design of a square MEMS piezoelectric accelerometer with a wide range of applicability, a low transverse sensitivity ratio, and high accuracy. IEEE Sens. J. **22**(10), 9306–9312 (2022). <https://doi.org/10.1109/JSEN.2022.3161671>
25. S. Li, X. Liang, W. Cheng, C. Zhen, D. Hu et al., Design and prototyping of a combined sensor for vibration and temperature measurement in high-temperature environments. IEEE Trans. Instrum. Meas. **73**, 9512809 (2024). <https://doi.org/10.1109/TIM.2024.3406773>
26. Y. Liu, B. Hu, Y. Cai, W. Liu, A. Tovstopyat et al., A novel tri-axial piezoelectric MEMS accelerometer with folded beams. Sensors **21**(2), 453 (2021). <https://doi.org/10.3390/s21020453>
27. C. Saayujya, J.S. Tan, Y. Yuan, Y.-R. Wong, H. Du, Design, fabrication and characterization of a zinc oxide thin-film piezoelectric accelerometer. 2014 IEEE Ninth International Conference on Intelligent Sensors, Sensor Networks and Information Processing (ISSNIP)., 1–6. IEEE (2014).
28. S. Shanmugavel, K. Yao, T.D. Luong, S.R. Oh, Y. Chen et al., Miniaturized acceleration sensors with in- plane polarized piezoelectric thin films produced by micromachining. IEEE Trans. Ultrason. Ferroelectr. Freq. Control **58**(11), 2289–2296 (2011). <https://doi.org/10.1109/TUFFC.2011.2086>
29. S. Trivedi, T. Shen, C.-Y. Chang, P.-W. Huang, S.-S. Li, Design of piezoelectric MEMS accelerometer module and its application in surface roughness prediction of fused silica substrate. IEEE Sens. J. **21**(19), 21979–21988 (2021). <https://doi.org/10.1109/JSEN.2021.3103059>
30. C.-C. Tsai, Y.-C. Chien, C.-S. Hong, S.-Y. Chu, C.-L. Wei et al., Study of Pb(Zr_0.52_Ti_0.48_)O_3_ microelectromechanical system piezoelectric accelerometers for health monitoring of mechanical motors. J. Am. Ceram. Soc. **102**(7), 4056–4066 (2019). <https://doi.org/10.1111/jace.16255>
31. L.-P. Wang, R.A. Wolf, Y. Wang, K.K. Deng, L. Zou et al., Design, fabrication, and measurement of high-sensitivity piezoelectric microelectromechanical systems accelerometers. J. Microelectromech. Syst. **12**(4), 433–439 (2003). <https://doi.org/10.1109/jmems.2003.811749>
32. Y.-H. Wang, P. Song, X. Li, C. Ru, G. Ferrari et al., A paper-based piezoelectric accelerometer. Micromachines **9**(1), 19 (2018). <https://doi.org/10.3390/mi9010019>
33. H. Wei, W. Geng, K. Bi, T. Li, X. Li et al., High-performance piezoelectric-type MEMS vibration sensor based on LiNbO(3) single-crystal cantilever beams. Micromachines **13**(2), 329 (2022). <https://doi.org/10.3390/mi13020329>
34. G.-D. Wu, X.-L. Liu, F.-P. Yu, F.-L. Li, S.-W. Tian et al., The accelerometer utilizing the transverse vibration mode of LGT piezoelectric crystal. 2019 13th Symposium on Piezoelectrcity, Acoustic Waves and Device Applications (SPAWDA). January 11-14, 2019. Harbin, China. IEEE, (2019).: 1-5. <https://doi.org/10.1109/spawda.2019.8681812>
35. G. Wu, Q. Yao, X. Liu, F. Yu, X. Zhao, A piezoelectric vibration sensor with excellent performance based on Bi_12_SiO_2_0 crystal for high-temperature applications. Appl. Phys. Lett. **125**, 012901 (2024). <https://doi.org/10.1063/5.0211683>
36. B. Yaghootkar, S. Azimi, B. Bahreyni, A high-performance piezoelectric vibration sensor. IEEE Sens. J. **17**(13), 4005–4012 (2017). <https://doi.org/10.1109/JSEN.2017.2707063>
37. J. Yang, M. Zhang, Y. He, Y. Su, G. Han et al., A resonant Z-axis aluminum nitride thin-film piezoelectric MEMS accelerometer. Micromachines **10**(9), 589 (2019). <https://doi.org/10.3390/mi10090589>
38. J. Yang, M. Zhang, C. Si, G. Han, J. Ning et al., A T-shape aluminum nitride thin-film piezoelectric MEMS resonant accelerometer. J. Microelectromech. Syst. **28**(5), 776–781 (2019). <https://doi.org/10.1109/JMEMS.2019.2924956>
39. C. Yang, B. Hu, L. Lu, Z. Wang, W. Liu et al., A miniaturized piezoelectric MEMS accelerometer with polygon topological cantilever structure. Micromachines **13**(10), 1608 (2022). <https://doi.org/10.3390/mi13101608>
40. H. Yang, Y. Sun, H. Gao, X. Zhou, H. Tan et al., Lead-free BF–BT ceramics with ultrahigh curie temperature for piezoelectric accelerometer. IEEE Trans. Ultrason., Ferroelect., Freq. Contr. **69**(11), 3102–3107 (2022). <https://doi.org/10.1109/tuffc.2022.3143575>
41. Z. Zhang, L. Zhang, Z. Wu, Y. Gao, L. Lou, A high-sensitivity MEMS accelerometer using a Sc(0.8)Al(0.2)N-based four beam structure. Micromachines **14**(5), 1069 (2023). <https://doi.org/10.3390/mi14051069>
42. Y. Zheng, X. Tu, J. Chen, P. Gao, E. Shi, Piezoelectric acceleration sensors based on LGX and ReCOB crystals for application above 645ºC. 2013 Joint European Frequency and Time Forum & International Frequency Control Symposium (EFTF/IFC)., 977–979. IEEE (2014).
43. H. Zhang, X. Li, X. Qiao, H. Wei, K. Bi et al., High sensitivity MEMS vibration sensors based on LiNbO_3_ ferroelectric single-crystal films. Ceram. Int. **51**(16), 21810–21819 (2025). <https://doi.org/10.1016/j.ceramint.2025.02.342>
44. Z. Fan, X. Yang, Y. Sun, Y. Guo, Y. Cui, High-g acceleration measurement for layer-counting tests based on coupling piezoelectric effect of PVDF film. Measurement **256**, 118471 (2025). <https://doi.org/10.1016/j.measurement.2025.118471>
45. C. Zhang, Y. He, Q. Wang, W. Pan, Z. Xiang et al., Self-assembled triboelectric nanogenerators with optimized surfaces for high-g accelerometers in extreme environments. Chem. Eng. J. **522**, 167545 (2025). <https://doi.org/10.1016/j.cej.2025.167545>
46. H. Zhao, X. Liu, Y. Tang, A. Kim, Y. Li, A novel compressible piezocomposite design for acceleration and dynamic force sensing. Mater. Des. **257**, 114492 (2025). <https://doi.org/10.1016/j.matdes.2025.114492>
